# Nucleic Acid-Sensing and Interferon-Inducible Pathways Show Differential Methylation in MZ Twins Discordant for Lupus and Overexpression in Independent Lupus Samples: Implications for Pathogenic Mechanism and Drug Targeting

**DOI:** 10.3390/genes12121898

**Published:** 2021-11-26

**Authors:** Miranda C. Marion, Paula S. Ramos, Prathyusha Bachali, Adam C. Labonte, Kip D. Zimmerman, Hannah C. Ainsworth, Sarah E. Heuer, Robert D. Robl, Michelle D. Catalina, Jennifer A. Kelly, Timothy D. Howard, Peter E. Lipsky, Amrie C. Grammer, Carl D. Langefeld

**Affiliations:** 1 Department of Biostatistics and Data Science, Division of Public Health Sciences, Wake Forest School of Medicine, Winston-Salem, NC 27157, USA; mimarion@wakehealth.edu (M.C.M.); hainswor@wakehealth.edu (H.C.A.); 2 Center for Precision Medicine, Wake Forest School of Medicine, Winston-Salem, NC 27157, USA; kdzimmer@wakehealth.edu; 3 Division of Rheumatology and Immunology, Department of Medicine, Medical University of South Carolina, Charleston, SC 29425, USA; ramosp@musc.edu; 4 AMPEL BioSolutions, LLC and RILITE Research Institute, Charlottesville, VA 22902, USA; prathyusha.bachali@ampelbiosolutions.com (P.B.); adam.labonte@ampelbiosolutions.com (A.C.L.); sarah.heuer@tufts.edu (S.E.H.); robert.robl@ampel.org (R.D.R.); michellecatalina@ampel.org (M.D.C.); peterlipsky@ampelbiosolutions.com (P.E.L.); amriegrammer@ampelbiosolutions.com (A.C.G.); 5 The Jackson Laboratory, Tufts Graduate School of Biomedical Sciences, Bar Harbor, ME 04609, USA; 6 Arthritis and Clinical Immunology Research Program, Oklahoma Medical Research Foundation, Oklahoma City, OK 73104, USA; Jennifer-Kelly@omrf.org; 7 Department of Biochemistry, Wake Forest School of Medicine, Winston-Salem, NC 27157, USA; tdhoward@wakehealth.edu

**Keywords:** epigenetics, nucleic acid sensing, methylation, gene expression, SLE, drug repositioning, RIG-I, lupus

## Abstract

Systemic lupus erythematosus (SLE) is a chronic, multisystem, autoimmune inflammatory disease with genomic and non-genomic contributions to risk. We hypothesize that epigenetic factors are a significant contributor to SLE risk and may be informative for identifying pathogenic mechanisms and therapeutic targets. To test this hypothesis while controlling for genetic background, we performed an epigenome-wide analysis of DNA methylation in genomic DNA from whole blood in three pairs of female monozygotic (MZ) twins of European ancestry, discordant for SLE. Results were replicated on the same array in four cell types from a set of four Danish female MZ twin pairs discordant for SLE. Genes implicated by the epigenetic analyses were then evaluated in 10 independent SLE gene expression datasets from the Gene Expression Omnibus (GEO). There were 59 differentially methylated loci between unaffected and affected MZ twins in whole blood, including 11 novel loci. All but two of these loci were hypomethylated in the SLE twins relative to the unaffected twins. The genes harboring these hypomethylated loci exhibited increased expression in multiple independent datasets of SLE patients. This pattern was largely consistent regardless of disease activity, cell type, or renal tissue type. The genes proximal to CpGs exhibiting differential methylation (DM) in the SLE-discordant MZ twins and exhibiting differential expression (DE) in independent SLE GEO cohorts (DM-DE genes) clustered into two pathways: the nucleic acid-sensing pathway and the type I interferon pathway. The DM-DE genes were also informatically queried for potential gene–drug interactions, yielding a list of 41 drugs including a known SLE therapy. The DM-DE genes delineate two important biologic pathways that are not only reflective of the heterogeneity of SLE but may also correlate with distinct IFN responses that depend on the source, type, and location of nucleic acid molecules and the activated receptors in individual patients. Cell- and tissue-specific analyses will be critical to the understanding of genetic factors dysregulating the nucleic acid-sensing and IFN pathways and whether these factors could be appropriate targets for therapeutic intervention.

## 1. Introduction

Systemic lupus erythematosus (SLE) is a chronic and severe systemic autoimmune disease characterized by the over-production of autoantibodies and heterogeneous clinical manifestations. With more than 100 risk loci identified, a genetic etiology for SLE is unequivocal [1,2,3,4,5,6,7,8]. In fact, the cumulative effect of these risk loci is substantial; the odds ratio (OR) for SLE in individuals of European ancestry is 30 when comparing individuals with the highest 10% of risk allele genetic load (i.e., polygenetic risk score—the weighted count of the number of risk alleles) to individuals in the lowest 10% of genetic load [6]. Despite the strong genetic contribution to risk, the concordance rate between monozygotic (MZ) twins ranges between 24–35%, suggesting that much of the risk remains unexplained and highlighting the potential importance of epigenetic and environmental factors in SLE susceptibility [9].

There is compelling evidence that epigenetic mechanisms, such as 5’ Cytosine methylation, are involved in the pathogenesis of SLE. For example, promoter demethylation at multiple genes in T cells treated with DNA demethylating agents are sufficient to cause lupus in animal models [10]. In recent years, several studies have investigated DNA methylation in SLE patients on a genome-wide scale. The earliest of these genome-wide studies interrogated 27,578 CpG sites in 12 SLE patients and 12 healthy controls using the Illumina Infinium HumanMethylation27 Beadchip, and identified 336 differentially methylated genes, the majority of which were hypomethylated in the cases relative to the controls [11]. Subsequent studies have examined genome-wide methylation in larger samples of SLE patients using the HumanMethylation450 Beadchip (>485,000 CpG sites) in a number of cell types, including naïve CD4+ T cells [12,13,14,15,16], memory and regulatory T cells [17], CD19+ B cells [17], CD14+ monocytes [14,17], granulocytes [14], neutrophils [18], and whole blood or peripheral blood mononuclear cells (PBMC) [19,20,21,22,23,24,25]. Differential methylation has not only been observed when comparing SLE patients to healthy controls, but similar patterns have been identified in SLE patients with nephritis [12,19,22], skin involvement [13], specific antibodies [20], and pediatric SLE [26]. The primary and consistent finding across all these studies has been hypomethylation of interferon-regulated genes across various cell types in cases, regardless of SLE disease activity [27].

The analysis of phenotypically discordant MZ twins represents the ideal design by which to assess the role of epigenetic variation in disease etiology and trait heritability while controlling for genetic background [28] and has revealed the existence of differentially methylated regions associated with several autoimmune diseases, including SLE [29], type 1 diabetes [30], psoriasis [31], and ulcerative colitis [32]. To date, the only previously published twin methylation study in SLE that exclusively used MZ twins quantified DNA methylation in white blood cells from 15 discordant MZ twin pairs at 1505 CpG sites in 807 genes using the Illumina GoldenGate Methylation Cancer Panel I [29]. Here, we performed a genome-wide analysis of DNA methylation in a discovery cohort of MZ twins discordant for SLE. The discovery cohort consisted of three twin pairs of European descent, and methylation was measured in whole blood using Illumina’s HumanMethylation450 Beadchip. The two strongest associated signals were validated using pyrosequencing. Findings from the discovery cohort were replicated in an independent set of MZ twins from Denmark. We then evaluated gene expression data from multiple cell types and kidney biopsies from 10 independent SLE cohorts to identify genes proximal to CpGs exhibiting differential methylation (DM) in the SLE-discordant MZ twins and exhibiting differential expression (DE) in independent SLE GEO cohorts (DM-DE genes) for pathway analyses. Together, the methylation, gene expression, and pathway analyses uncovered two separable yet complimentary molecular pathways of lupus pathogenesis, shedding light on potential drug repositioning opportunities and novel therapeutic targets for SLE.

## 2. Materials and Methods

### 2.1. Discovery Cohort

Genomic DNA was extracted from peripheral blood of three female MZ twin pairs of European ancestry discordant for SLE enrolled in the Lupus Family Registry and Repository (LFRR) [33]. All cases met ACR classification criteria for SLE [34].

### 2.2. Replication Cohort

An SLE study of 15 twin pairs from Denmark, assayed on the HumanMethylation450 Beadchip, in monocytes, CD4+ T cells, CD19+ B cells, and granulocytes, was published in 2018 by Ulff-Moller et al. [14]. These data were downloaded from the Gene Expression Omnibus (GEO, accession no. GSE110607), and all available female MZ twin pairs discordant for SLE were retained for analysis (4 twin pairs). The publication states that of these four female MZ twin pairs discordant for SLE, two of the non-SLE twins had other autoimmune diseases, including Sjogren’s syndrome, systemic sclerosis, autoimmune thyroiditis, and primary biliary cirrhosis. However, this clinical information was not available in GEO.

### 2.3. Genome-Wide DNA Methylation Assay and Array Validation in LFRR Twins

Genomic DNA (1μg) from each individual was treated with sodium bisulfite using the EZ 96-DNA methylation kit (Zymo Research, Irvine, CA, USA), following the manufacturer’s standard protocol. Genome-wide DNA methylation was assessed using the Illumina Infinium HumanMethylation450 BeadChip (Illumina, Inc., San Diego, CA, USA), which interrogates over 485,500 CpG sites that cover 99% of RefSeq genes (including the promoter, 5’UTR, first exon, gene body, and 3’UTR), as well as 96% of CpG islands and island shores. Arrays were processed using the manufacturer’s standard protocol, with both members of each twin pair being hybridized to the same row on the microarray to minimize batch effects. GenomeStudio software (Illumina, Inc.) was used to perform initial quality control and to calculate the relative methylation level of each interrogated cytosine, which is reported as a β-value given by the ratio of the normalized signal from the methylated probe to the sum of the normalized signals of the methylated and unmethylated probes. This β-value for each CpG site ranges from 0 (unmethylated) to 1 (fully methylated). CpG loci with a stringent detection *p*-value > 1.0 × 10^−5^ in any of the samples were excluded (*n* = 2118 probes) to control for poor-quality assays. Validation of the array data in the LFRR twins was performed by pyrosequencing two of the most significant CpGs probes: cg13304609 (in IFI44L) and cg23570810 (in IFITM1). The correlations between the methylation proportions from the array and pyrosequencing for these two probes were *r*^2^ = 0.98 and *r*^2^ = 0.99, respectively.

### 2.4. Collection of Gene Expression Experiments from SLE Patient Datasets

Raw data were downloaded from 10 publicly available gene expression datasets (Supplemental Table S1). Only datasets from female lupus patients were analyzed. Active SLE was defined as a Systemic Lupus Erythematosus Disease Activity Index (SLEDAI) > 6 [35]. This has become the standard threshold for disease activity in recent clinical trials of SLE. 

### 2.5. Data Analysis

To identify differentially methylated genes between unaffected and SLE-affected twins, a paired t-test on the probe-specific β-values was computed separately for the discovery and replication twin datasets. For the discovery set, CpG sites meeting (1) the Benjamini–Hochberg False Discovery Rate (FDR) [36] threshold P*_FDR_* < 0.05 (equivalent to *p* < 1.06 × 10^−7^) and (2) a mean DNA methylation difference of (Δ*β*) > |0.085| were considered statistically significant; the mean methylation difference threshold was obtained by maximizing the area under the receiver operator characteristic curve (AUC) as a function of the β-value (described below). The genes related to the differentially methylated CpG sites (as annotated by Illumina for the HumanMethylation450) were queried in the Interferome online database to identify interferon-regulated genes [37]. In addition, significant CpG sites were investigated for evidence of association between DNA methylation pattern and gene expression (mQTL) using the iMETHYL genome browser [38]. These results are based on 100 healthy subjects with RNA-seq data and DNA methylation data in CD4T cells, monocytes, and PBMC.

Statistical analysis of the expression data was completed using the following R packages available from Bioconductor: GEOquery, affy, affycoretools, simpleaffy, gcrma, LIMMA, and GSVA. Non-normalized arrays were first inspected for visual artifacts and poor RNA hybridization using Affymetrix QC plots. Principal component (PC) plots were generated for all cell types in each experiment to identify outliers. After removing outliers, the datasets were normalized using the gcrma package (available in Bioconductor [39], www.bioconductor.org) resulting in log2 intensity values for the R expression set objects (denoted E-sets); an E-set combines several information types in a single structured object: an expression value matrix, phenotypic metadata corresponding to individual samples (phenoData), annotation data describing each feature (probeset) of a microarray platform (featureData), as well as other separate metadata matrices describing the experimental protocol and array platform design. To increase the probability of identifying differentially expressed genes (DE genes), the analyses were completed using normalized datasets prepared using both the native Affymetrix chip definition file (CDF), as well as custom BrainArray Entrez CDFs. Illumina CDFs were used for GSE49454.

The CDF-annotated E-sets were filtered to remove probes with very low intensity values by computing the mean log2 values for each probe across all samples and removing those in the lower half of the range of mean values from the expression set (E-set). Probes missing gene annotation data were also discarded. GCRMA normalized expression values were variance-corrected using local empirical Bayesian shrinkage before calculation of differential expression using the ebayes function in the Bioconductor limma package [40]. The resulting p-values were adjusted for multiple hypothesis testing using Benjamini–Hochberg False Discovery Rate (FDR) [36]. Significant Affymetrix and BrainArray probes within each study were merged and filtered to retain DE probes with a PFDR < 0.2. This list was filtered to retain only the most significant probe per gene. 

**Figure 1:**
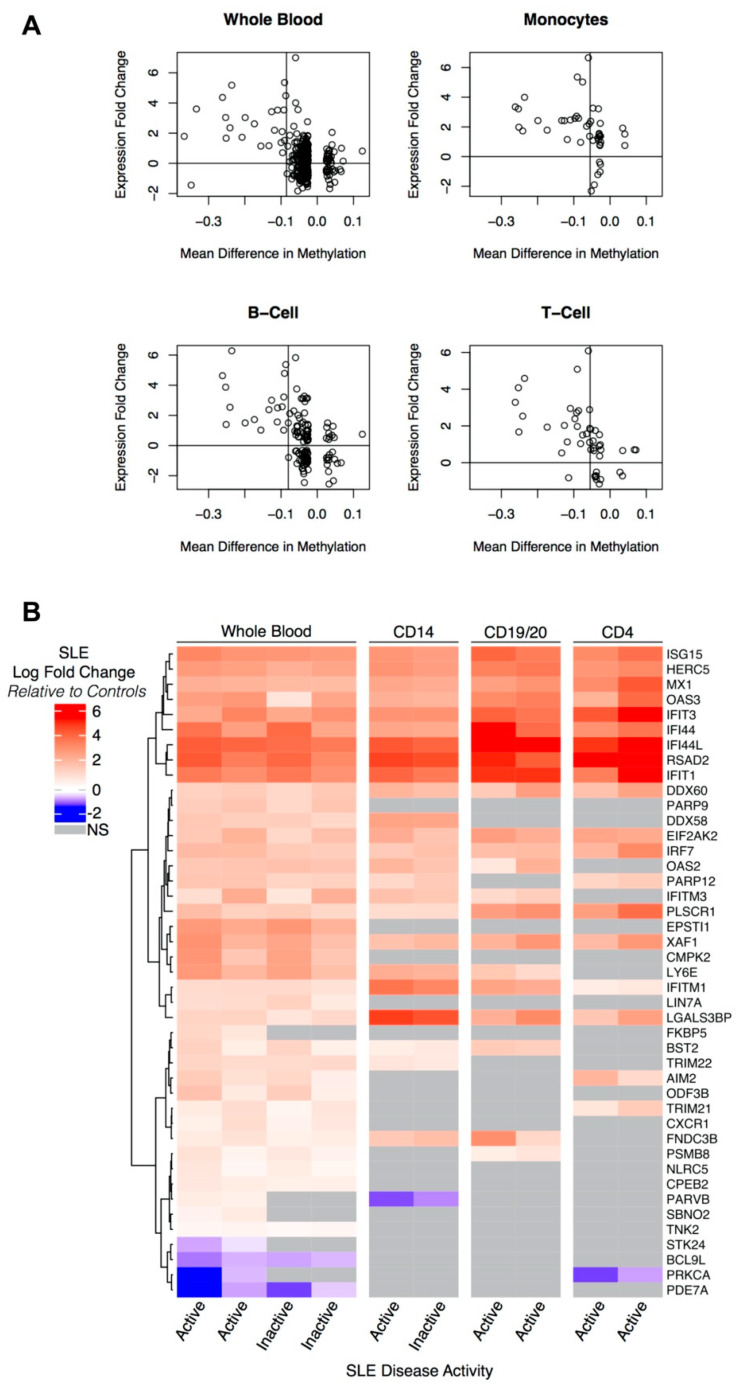
Hypomethylated genes showing differential expression in independent SLE cohorts. (**A**) Specific thresholds for the difference in the β-value (from the discordant twin methylation experiment in whole blood) that maximize the area under the ROC curve predicting increased differential expression in the independent SLE whole blood experiments (GSE39088, GSE49454), monocytes (GSE38351), B-cells (GSE10325, GSE4588), and T cells (GSE10325, GSE51997) are shown as vertical bars. Genes with differential methylation *p*-values greater than 0.0001 and a mean DNA methylation difference of (Δ*β*) > |0.025| have been removed from the plots. (**B**) Heatmap of 43 genes hypomethylated in the discordant twin data (∆β < −0.085) and differentially expressed between controls and active (SLEDAI ≥ 6) or inactive (SLEDAI < 6) lupus patients from two whole blood experiments, monocytes, B cells, and T cells. Hierarchical clustering was performed across rows with Euclidean distance metric and complete linkage. Blue/red gradient represents the log fold change values in lupus patients compared to controls.

To identify DM-DE genes, we used a logistic regression model (expression fold change as a binary outcome > 0 versus < 0) to determine cell-type specific thresholds for the difference in the β-value that maximized the area under the ROC curve (AUC) predicting increased differential expression (Figure 1A, Supplemental Figure S1). These thresholds were determined by calculating the area under the receiver operating characteristic curve (AUC) across points at regular intervals between 0 and −0.15 and selecting the values that maximized the AUC. Primary inferences are based on thresholds, which included a logFC in expression > 0 and a mean difference in β < −0.085, −0.055, −0.08, and −0.055 in whole blood, monocytes, B cells, and T cells, respectively. Figure 1A displays these thresholds as vertical bars. For clarity, genes with differential methylation *p*-values greater than 0.0001 and a mean DNA methylation difference of (Δ*β*) > |0.025| have been removed from Figure 1A.

The DM-DE genes were analyzed in a pathway analysis using the MCODE [41] clustering algorithm and STRING networking scores [42].

Protein–drug interaction networks were generated for each DM-DE gene individually via STITCH [43], Ingenuity Pathway Analysis (IPA) (Qiagen Bioinformatics: ingenuity.com), and the Drug–Gene Interaction database [44]. Drugs were denoted as (1) known utility in lupus therapy, (2) FDA-approved compound, (3) currently involved in a clinical trial (not necessarily SLE), and (4) generally regarded as safe (GRAS) compounds. Using a hypothesis-driven ranking of the therapeutic potential for SLE applications of specific drugs or compounds, the combined lupus treatment scoring (CoLTS) scores (range −16 to +11) were calculated [45].

## 3. Results

### 3.1. Characteristics of the MZ Twins

The LFRR MZ twins were all females of European ancestry, and the SLE-diagnosed twins exhibited a range of SLE clinical conditions (Supplemental Table S2). The Danish MZ twins were also all females of European ancestry. Clinical characteristics such as number of ACR criteria, SLEDAI score, autoantibodies, and medications are described in Ulff-Moller et. al., but were not available in GEO [14].

### 3.2. Identification of Differentially Methylated Regions in Twins Discordant for SLE

Of the 485,577 CpG sites passing quality control metrics, 59 sites in 33 genes met both a P*_FDR_* < 0.05 (equivalent to a non-FDR *p* < 1.06 × 10^−7^) and a mean DNA methylation difference of (Δ*β*) > |0.085| (Table 1). Only two of these significant CpG sites showed increased methylation in the affected twins (hypermethylation), while the remaining 57 exhibited lower methylation (hypomethylation). Of the 33 genes represented in Table 1, 22 are regulated at some level by type I interferons (as defined by Interferome [37]). Eleven genes are novel to our study and have not been previously reported as SLE-related in a genome-wide methylation study, five of which are unrelated to the typical interferon signature (*LY6G5C, CXCR1, ATOH8, CACNA1D, MECOM*). Lymphocyte antigen 6 complex, locus G5C (*LY6G5C*), is located within the major histocompatibility complex class III region and codes for a protein associated with the cell membrane by a glycosylphosphatidylinositol linkage and involved in signal transduction [46]. Chemokine (C-X-C motif) receptor 1 (*CXCR1*) encodes for a protein that is a receptor for interleukin 8. Genetic and expression variation in *CXCR1* have been correlated with infections (e.g., active tuberculosis, hepatitis B, *Candida albicans*) and modestly with SLE [6,47,48,49,50]. Atonal bHLH transcription factor 8 (*ATOH8*), calcium voltage-gated channel subunit alpha1 D (*CACNA1D*), and MDS1, and EVI1 complex locus (*MECOM*) do not have known links to autoimmune disease or infections. Given the gender bias in SLE, it is interesting to note that none of the differentially methylated probes meeting our significance criteria were located on the X chromosome.

We next examined the 59 differentially methylated CpGs from the discovery cohort (Table 1) in the Danish twin replication cohort. Even with the probable dampening effect generated by two of the Danish non-SLE twins having other autoimmune diseases, we observed very high concordance in the direction of the Δ*β* values. Specifically, 55 (93%), 54 (92%), 52 (88%), and 54 (92%) of the 59 differentially methylated CpG sites in the LFRR twins were concordant in the Danish twins’ monocytes, CD4+ T cells, CD19+ B cells, and granulocytes, respectively. Furthermore, 35, 26, 32, and 33 of the 59 CpG sites were statistically significant (*p*-value < 0.05) and directionally concordant in the monocyte, CD4+ T cell, CD19+ B cell, and granulocyte expression datasets, respectively; only one of these was statistically significant in the opposite direction (*p*-value < 0.05; Additional File 1). Thus, the Danish twin data strongly corroborated the global pattern of methylation observed in the LFRR twin data. 

**Table 1:** Differentially methylated probes from three monozygotic twin pairs discordant for SLE.

CpG *	Chr	Pos (bp) ^†^	Gene	Δ*β*				*p*-Value	Interferon-Regulated ^‡^	Relation to CpG ^††^
Pair1	Pair2	Pair3	Mean
cg13304609	1	79085162	*IFI44L*	−0.24	−0.27	−0.37	−0.29	1.58 × 10^−14^	IRG	
cg06872964	1	79085250	*IFI44L*		−0.26	−0.21	−0.24	1.05 × 10^−71^	IRG	
cg03607951	1	79085586	*IFI44L*	−0.27	−0.3	−0.21	−0.26	7.23 × 10^−22^	IRG	
cg17515347	1	159047163	*AIM2*	−0.09	−0.11	−0.07	−0.09	3.01 × 10^−12^	IRG	
cg08272268	1	200380059	*ZNF281*	−0.08	−0.07	−0.11	−0.09	4.33 × 10^−15^		S_Shore
cg01028142	2	7004578	*CMPK2*	−0.22	−0.36	−0.43	−0.33	7.98 × 10^−8^	IRG	N_Shore
cg10959651	2	7018020	*RSAD2*	−0.13	−0.1	−0.16	−0.13	3.14 × 10^−14^	IRG	
cg10549986	2	7018153	*RSAD2*	−0.08	−0.09	−0.1	−0.09	1.95 × 10^−91^	IRG	
cg14126601	2	37384708	*EIF2AK2*	−0.08	−0.1	−0.12	−0.1	5.55 × 10^−16^	IRG	S_Shore
cg26337070	2	85999873	*ATOH8*	−0.06	−0.12	−0.11	−0.1	7.55 × 10^−9^		
cg04781494	2	202047246	*CASP10*	−0.07	−0.13	−0.08	−0.09	8.39 × 10^−8^	IRG	
cg15768138	2	219030752	*CXCR1*	−0.09	−0.12	−0.11	−0.11	7.38 × 10^−27^		
cg13411554	3	53700276	*CACNA1D*	−0.06	−0.12	−0.09	−0.09	8.66 × 10^−8^		
cg22930808	3	122281881	*PARP9-DTX3L*	−0.36	−0.34	−0.4	−0.37	6.74 × 10^−126^	IRG	N_Shore
cg08122652	3	122281939	*PARP9-DTX3L*	−0.34	−0.31	−0.51	−0.38	1.11 × 10^−9^	IRG	N_Shore
cg00959259	3	122281975	*PARP9-DTX3L*	−0.37	−0.3	−0.34	−0.34	1.32 × 10^−56^	IRG	N_Shore
cg06981309	3	146260954	*PLSCR1*	−0.24	−0.28	−0.21	−0.24	6.41 × 10^−31^	IRG	N_Shore
cg02556393	3	168866705	*MECOM*	−0.08	−0.09	−0.1	−0.09	3.14 × 10^−95^		N_Shore
cg07809027	4	15007205	*CPEB2*	−0.07	−0.1	−0.12	−0.1	2.08 × 10^−14^		S_Shore
cg02215171	4	89379156	*HERC5*	−0.08	−0.09	−0.11	−0.09	4.48 × 10^−18^	IRG	S_Shore
cg17786255	4	108814389	*SGMS2*	−0.07	−0.09	−0.11	−0.09	2.01 × 10^−16^	IRG	
cg21873524	4	190942744		−0.1	−0.1	−0.12	−0.11	1.03 × 10^−55^		Island
cg24740632	5	134486678		−0.11	−0.12	−0.14	−0.12	2.26 × 10^−60^		
cg06012695	6	28770593			−0.1	−0.13	−0.11	3.59 × 10^−16^		
cg25138053	6	31368016		−0.11	−0.09	−0.07	−0.09	3.67 × 10^−15^		S_Shore
cg22708150	6	31649619	*LY6G5C*	−0.12	−0.14	−0.17	−0.14	1.05 × 10^−19^		N_Shore
cg07292773	6	156718177		0.07	0.1	0.11	0.1	2.22 × 10^−17^		Island
cg12013713	7	139760671	*PARP12*	−0.12	−0.14	−0.09	−0.12	1.44 × 10^−16^	IRG	N_Shore
cg20190772	8	48572496	*KIAA0146*	−0.08	−0.07	−0.13	−0.09	1.40 × 10^−8^		
cg14864167	8	66751182	*PDE7A*	−0.25	−0.35	−0.45	−0.35	1.21 × 10^−9^		N_Shelf
cg06102678	8	81491328		−0.08	−0.12	−0.07	−0.09	1.00 × 10^−8^		Island
cg12110437	8	144098888	*LY6E*	−0.16	−0.17	−0.27	−0.2	3.14 × 10^−9^	IRG	N_Shore
cg17555806	10	74448117		−0.08	−0.12	−0.07	−0.09	1.51 × 10^−8^		N_Shelf
cg02314339	10	91020653		−0.08	−0.14	−0.11	−0.11	1.72 × 10^−8^		
cg06188083	10	91093005	*IFIT3*	−0.29	−0.16	−0.31	−0.25	6.18 × 10^−8^	IRG	
cg05552874	10	91153143	*IFIT1*	−0.2	−0.28	−0.3	−0.26	6.01 × 10^−16^	IRG	
cg14910175	10	131840954		−0.07	−0.11	−0.08	−0.09	1.56 × 10^−11^		N_Shelf
cg10552523	11	313478	*IFITM1*	−0.14	−0.12	−0.14	−0.13	5.90 × 10^−115^	IRG	N_Shelf
cg20566897	11	313527	*IFITM1*	−0.11	−0.11	−0.09	−0.1	7.00 × 10^−62^	IRG	N_Shelf
cg23570810	11	315102	*IFITM1*	−0.24	−0.25	−0.34	−0.27	1.43 × 10^−18^	IRG	N_Shore
cg03038262	11	315262	*IFITM1*	−0.24	−0.22	−0.29	−0.25	4.41 × 10^−40^	IRG	N_Shore
cg20045320	11	319555		−0.19	−0.13	−0.2	−0.18	4.85 × 10^−17^		S_Shore
cg17990365	11	319718	*IFITM3*	−0.16	−0.15	−0.15	−0.16	8.78 × 10^−295^	IRG	S_Shore
cg08926253	11	614761	*IRF7*	−0.15	−0.14	−0.23	−0.17	2.01 × 10^−9^	IRG	Island
cg12461141	11	5710654	*TRIM22*	−0.1	−0.08	−0.12	−0.1	6.35 × 10^−25^	IRG	
cg23571857	17	6658898	*XAF1*	−0.07	−0.13	−0.11	−0.1	1.46 × 10^−8^	IRG	
cg04927537	17	76976091	*LGALS3BP*	−0.14	−0.11	−0.2	−0.15	2.77 × 10^−10^	IRG	
cg25178683	17	76976267	*LGALS3BP*	−0.15	−0.11	−0.21	−0.16	2.01 × 10^−8^	IRG	
cg16503797	18	19476805		−0.08	−0.12	−0.08	−0.09	5.39 × 10^−12^		N_Shore
cg15871086	18	56526595		−0.07	−0.11	−0.08	−0.09	2.08 × 10^−11^		N_Shelf
cg23352030	20	62198469	*PRIC285*	0.13	0.19	0.11	0.14	2.36 × 10^−11^		Island
cg16785077	21	42791867	*MX1*	−0.11	−0.09	−0.12	−0.11	8.45 × 10^−27^	IRG	N_Shore
cg22862003	21	42797588	*MX1*	−0.31	−0.25	−0.35	−0.31	1.62 × 10^−25^	IRG	N_Shore
cg26312951	21	42797847	*MX1*	−0.26	−0.17	−0.2	−0.21	6.28 × 10^−15^	IRG	N_Shore
cg21549285	21	42799141	*MX1*	−0.5	−0.35	−0.57	−0.47	6.59 × 10^−13^	IRG	S_Shore
cg05543864	22	24979755	*GGT1*	−0.08	−0.08	−0.1	−0.09	1.44 × 10^−45^		
cg20098015	22	50971140	*ODF3B*	−0.19	−0.22	−0.21	−0.21	9.88 × 10^−83^	IRG	S_Shore
cg05523603	22	50973101		−0.17	−0.23	−0.27	−0.22	5.51 × 10^−14^		S_Shelf
cg02247863	22	50983415		−0.07	−0.1	−0.11	−0.09	2.51 × 10^−13^		N_Shore

* CpGs meeting the P_FDR_ < 0.05 threshold (equivalent to *p* < 1.06 × 10^−7^) and having |Δ*β|* > 0.085. ^†^ Positions are from Build 37. ^‡^ IRG as defined by Interferome [37]. ^††^ Island: CpG sites > 200 bp, with GC content > 55% and observed to expected ratio > 0.6. N_shore: 0–2 kb upstream from island; S-shore 0–2 kb downstream from island; N_shelf 2–4 kb upstream from island; S_shelf 2–4 kb downstream from island.

We also sought to determine if the dominating presence of the interferon signature might have masked more modest signals from other individual (non-IFN) loci. After regressing out the mean β-value (methylation value) for the most significant CpG site in each interferon-regulated gene in Table 1 (as defined by Interferome [37]), no additional CpG sites across the genome met an FDR threshold of significance (P*_FDR_* > 0.05).

We considered the genomic context of the CpG sites showing aberrant methylation in the LFRR MZ twins. Here, a CpG island was defined as a cluster of CpG sites of greater than 200 bp, with GC content >55%, and the observed-to-expected (under mathematical independence of the Gs and Cs) ratio >0.6 [51]. Interestingly, out of 59 CpG sites differentially methylated, the majority (54%, *n* = 32) were located in a CpG shore (0–2 kb from island) or shelf (2–4 kb from island), whereas only 8% (*n* = 5) were located in a CpG island (Table 1). This is in contrast to the composition of the 450k chip in which about one third of the CpG sites reside in islands (Supplemental Figure S2). Notably, the only two hypermethylated CpG sites (relative to the unaffected twin) meeting our significance thresholds reside in CpG islands. 

### 3.3. Hypomethylated Genes Are Overexpressed in Independent Cohorts

Methylation at CpG sites influences gene expression. Thus, linking differential methylation to changes in gene expression by showing that the same genes were associated with SLE in both types of experiments (even in independent samples) would provide further evidence of the importance of these genes and could identify potential actionable mechanisms. 

Genes harboring a CpG site with Δ*β* < −0.085 and *p* < 0.01 (for differential methylation) were tested for differential expression in whole blood from two independent cohorts, each comparing SLE patients to healthy controls (GSE39088 and GSE49454) (Table 2). Relative to controls, overexpression was observed in both active and inactive SLE patients within almost all of these genes, and the level of expression was highly correlated within the gene expression experiments (experiment 1, *r* = 0.95; experiment 2, *r* = 0.99). *IFI44L, RADS2,* and *IFIT1* showed the highest fold changes and comparable increases in expression in active and inactive SLE patients; *IFI44L* is noteworthy as it has been reported to be predictive of SLE status relative to healthy controls and other autoimmune diseases [52]. Cohorts with expression data derived from monocytes (GSE38351), CD19+, and CD20+ B cells (GSE10325, GSE4588), and CD4+ T cells (GSE10325, GSE51997) reflected a consistent pattern of increased expression in genes meeting the mean (methylation) Δ*β* threshold of −0.085 (Figure 1B). Upon extending Δ*β* to <−0.055, the statistically appropriate threshold for detecting differential expression in monocytes and T cells in our dataset (see Methods), an additional 54 hypomethylated genes were evaluated in the gene expression datasets (Supplemental Table S3). Overall, the pattern of differential expression of hypomethylated genes was very similar across the cell subtypes examined (Figure 1B, Supplemental Table S3). Thus, the differential expression results in independent cohorts in multiple cell types provide a multi-omic, independent pseudo-replication, and translational interpretation of the methylation results (Table 2).

Hierarchical clustering (Euclidean distance, complete linkage) of the DM-DE genes using the log fold change (LFC) identified a cluster of nine genes with markedly higher LFC (Figure 1B). This cluster shows a consistent pattern across whole blood, monocytes, B cells, and T cells, as well as in both active and inactive SLE disease. In fact, the LFC remained largely consistent between active and inactive disease across all DM-DE genes. Exceptions to this pattern include FK506 binding protein 5 (*FKBP5*), parvin beta (*PARVB*), and strawberry notch homolog 2 (*SBNO2*) in whole blood, where there is upregulation in active patients and non-significant change in inactive patients. This pattern was not replicated in any of the individual cell types. 

**Table 2:** Differential expression of hypomethylated genes in whole blood from two independent SLE cohorts.

							Active SLE ^§^		Inactive SLE ^§^	
CpG *	Chr	Pos (bp) ^†^	Gene	Mean Δ*β*	Methylation *p*-Value	Interferon-Regulated ^‡^	Log FC Expt 1	Log FC Expt 2	Log FC Expt 1	Log FC Expt 2
cg16526047	1	949893	*ISG15*	−0.11	1.28 × 10^−4^	IRG	3.1	2.77	2.74	2.59
cg05696877	1	79088769	*IFI44L*	−0.3	6.60 × 10^−6^	IRG	3.98	3.8	3.64	3.4
cg01079652	1	79118191	*IFI44*	−0.34	5.34 × 10^−4^	IRG	3.54	2.53	3.7	2.33
cg17515347	1	159047163	*AIM2*	−0.09	3.01 × 10^−12^	IRG	1.39	0.86	1.08	0.49
cg01028142	2	7004578	*CMPK2*	−0.33	7.98 × 10^−8^	IRG	2.76	1.5	2.43	1.51
cg10959651	2	7018020	*RSAD2*	−0.13	3.14 × 10^−14^	IRG	4.04	3.32	3.76	3.04
cg14126601	2	37384708	*EIF2AK2*	−0.1	5.55 × 10^−16^	IRG	1.47	2.02	1.08	1.68
cg15768138	2	219030752	*CXCR1*	−0.11	7.38 × 10^−27^		0.43	0.96	0.38	0.66
cg08122652	3	122281939	*PARP9-DTX3L*	−0.38	1.11 × 10^−9^	IRG	1.36	1.56	1.07	1.55
cg06981309	3	146260954	*PLSCR1*	−0.24	6.41 × 10^−31^	IRG	1.77	1.25	1.38	1.07
cg02694620	3	172109284	*FNDC3B*	−0.11	3.80 × 10^−3^		0.57	0.82	0.41	0.52
cg15065340	3	195632915	*TNK2*	−0.16	4.04 × 10^−3^		0.22	0.31	0.2	0.25
cg07809027	4	15007205	*CPEB2*	−0.1	2.08 × 10^−14^		0.66	0.52	0.42	0.45
cg02215171	4	89379156	*HERC5*	−0.09	4.48 × 10^−18^	IRG	2.62	2.48	2.14	2.36
cg05883128	4	169239131	*DDX60*	−0.25	2.13 × 10^−5^	IRG	1.24	1.38	1.06	1.46
cg08099136	6	32811251	*PSMB8*	−0.11	1.43 × 10^−4^	IRG	−0.39	−0.13	NS	NS
cg00052684	6	35694245	*FKBP5*	−0.16	1.65 × 10^−3^		1.11	0.71	NS	NS
cg05994974	7	139761087	*PARP12*	−0.15	6.89 × 10^−5^	IRG	1.52	1.57	1.14	1.25
cg14864167	8	66751182	*PDE7A*	−0.35	1.21 × 10^−9^		−1.24	−0.41	−0.82	−0.23
cg12110437	8	144098888	*LY6E*	−0.2	3.14 × 10^−9^	IRG	2.66	1.92	2.43	1.7
cg03848588	9	32525008	*DDX58*	−0.1	4.34 × 10^−4^	IRG	1.48	1.3	1.32	1.07
cg06188083	10	91093005	*IFIT3*	−0.25	6.18 × 10^−8^	IRG	2.25	3.15	2.3	2.87
cg05552874	10	91153143	*IFIT1*	−0.26	6.01 × 10^−16^	IRG	3.39	2.94	3.42	2.81
cg23570810	11	315102	*IFITM1*	−0.27	1.43 × 10^−18^	IRG	1	1.03	1.03	0.81
cg17990365	11	319718	*IFITM3*	−0.16	8.78 × 10^−295^	IRG	0.92	2.23	0.71	2.13
cg08926253	11	614761	*IRF7*	−0.17	2.01 × 10^−9^	IRG	1.84	1.79	1.4	1.37
cg08577913	11	4415193	*TRIM21*	−0.1	1.74 × 10^−3^	IRG	0.56	0.93	0.28	0.75
cg12461141	11	5710654	*TRIM22*	−0.1	6.35 × 10^−25^	IRG	1.14	1	0.99	1.05
cg26811705	11	118781408	*BCL9L*	−0.09	1.64 × 10^−3^		−0.6	−0.35	−0.41	−0.32
cg19347790	12	81332050	*LIN7A*	−0.09	1.87 × 10^−4^		0.93	0.99	1.24	0.61
cg25800166	12	113375896	*OAS3*	−0.13	5.36 × 10^−5^	IRG	2.52	2.69	0.73	2.35
cg19371652	12	113415883	*OAS2*	−0.11	2.24 × 10^−5^	IRG	1.48	1.56	1.64	1.53
cg03753191	13	43566902	*EPSTI1*	−0.1	9.23 × 10^−5^	IRG	2.65	2.26	2.71	2.02
cg00246969	13	99159656	*STK24*	−0.11	6.26 × 10^−6^		0.81	0.32	0.66	0.36
cg07839457	16	57023022	*NLRC5*	−0.23	6.10 × 10^−6^	IRG	0.7	0.23	0.53	0.27
cg23571857	17	6658898	*XAF1*	−0.1	1.46 × 10^−8^	IRG	2.85	1.96	2.35	1.68
cg23378941	17	64361956	*PRKCA*	−0.11	6.89 × 10^−5^	IRG	−1.11	−0.3	NS	NS
cg25178683	17	76976267	*LGALS3BP*	−0.16	2.0 × 10^−8^	IRG	1.16	1.21	0.72	1.05
cg07573872	19	1126342	*SBNO2*	−0.15	2.77 × 10^−3^	IRG	0.38	0.58	NS	NS
cg07839313	19	17514600	*BST2*	−0.12	3.48 × 10^−3^	IRG	1.24	0.49	1.17	0.41
cg21549285	21	42799141	*MX1*	−0.47	6.59 × 10^−13^	IRG	2.12	2	1.86	1.79
cg19460508	22	44422195	*PARVB*	−0.1	1.64 × 10^−3^		0.54	0.39	NS	NS
cg20098015	22	50971140	*ODF3B*	−0.21	9.88 × 10^−83^	IRG	1.61	0.61	1.36	0.47

Differential gene expression values come from GSE39088 (Expt 1) and GSE49454 (Expt 2) in whole blood of lupus patients compared with controls. * CpGs with *p* < 0.01 and |Δ*β|* > 0.085. ^†^ Positions are from Build 37. ^‡^ As defined by Interferome [37]. ^§^ Active disease is defined as ≥6 on the Systemic Lupus Erythematosus Disease Activity Index (SLEDAI) [35].

Although only one of the three affected MZ twins in the discovery cohort had renal involvement, almost all of the genes mapping to differentially methylated CpG sites showed overexpression in both the kidney glomerulus and tubulointerstitium from independent lupus nephritis patients (Table 3). In the glomerulus, 28 genes were overexpressed, 2 were under expressed, and 14 were not significantly differentially expressed in lupus nephritis samples compared to healthy controls. In the tubulointerstitium, 27 were overexpressed, 5 under expressed, and 12 not significantly differentially expressed. *IFI44L*, *MX1*, and *IFI44* showed the highest levels of overexpression across the two tissues. The fold change was correlated between the two tissues (*r* = 0.66, *p* < 0.0001). 

Significant DNA methylation sites were further investigated for evidence of association between DNA methylation at a specific CpG site and gene expression (eQTM) using the iMETHYL genome browser with data on 100 healthy Japanese subjects with RNA-seq data and DNA methylation data in CD4T cells, monocytes, and PBMC [38] (Supplemental Table S4). Most of the CpGs from Table 1 that are identified in iMETHYL are eQTMs for the gene in which they reside. In contrast, some are eQTMs for additional genes of interest. For example, cg17515347 is in physical proximity to *AIM1*, which has an important role in T cell regulation in autoimmune diseases. However, this CpG site is also an eQTM for five other genes in CD4+ T cells (*TAGLN2, SLAMF8, DUSP23, PHYIN1 FCRL6*), several of which have established autoimmune disease connections. Transgelin-2 may help regulate activation and migration of B cells in lymph node follicles, exhibits increased expression in B cells from lymph nodes in SLE patients, and appears important in host defense [53,54]. SLAM family member 8 (*SLAMF8*) is a member of the SLAM family of genes of which several members have been associated with multiple autoimmune diseases [55]. FcR-like 6 (*FCRL6*), a receptor that binds to major histocompatibility complex (MHC) class II HLA-DR, is expressed in B cells and has a tyrosine-based immunoregulatory function [56,57]. Dual-specificity protein phosphate 23 (*DUSP23*) expression is reportedly higher in CD4+ T cells from SLE patients compared to healthy controls [58]. Thus, DNA methylation in these regions, and potentially others, may have a complex and multifaceted impact on autoimmunity. Annotation of cg20098015 on chromosome 22 is linked to Outer Dense Fiber of Sperm Tails 3 (*ODF3B*). However, this CpG is an eQTM for SCO2 homolog, mitochondrial and SCO cytochrome oxidase deficient homolog 2 (*SCO2*), and thymidine phosphorylase (*TYMP*), both involved in mitochondrial functions.

### 3.4. Pathway Analysis of DM-DE Genes

**Figure 2:**
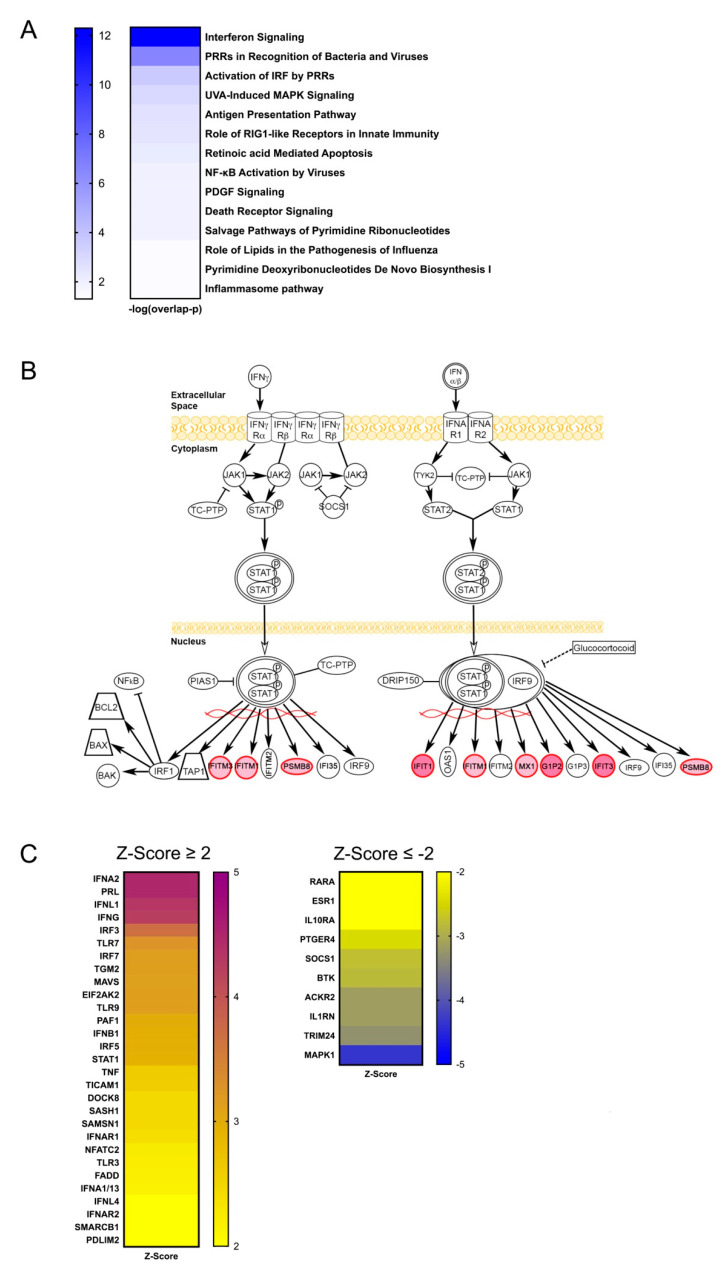
Pathway analyses of hypomethylated genes showing differential expression in independent SLE cohorts. (**A**) List and statistical significance of the overlap of the IPA canonical pathways comprised of hypomethylated genes showing differential expression in whole blood of independent SLE patients. (**B**) IPA canonical IFN signaling of hypomethylated genes showing differential expression (increased expression in SLE cases in red) in whole blood of independent SLE patients. (**C**) Activation Z-scores of genes predicted as upstream regulators of genes hypomethylated in the discordant twin data (∆β < −0.085) and differentially expressed in whole blood between independent SLE cases and controls. A positive (negative) Z-score indicates that a regulator has significantly more (fewer) activated predictions than inhibited predictions.

Pathway, clustering, and networking analyses were completed to elucidate patterns among the DM-DE genes. Ingenuity Pathway Analysis (IPA) identified two primary canonical pathways: (1) interferon signaling and (2) pattern recognition receptor (PRR) (Figure 2A). The overlap *p*-value, which tests for independence between known targets of each transcription regulator in a pathway and the list of genes provided, shows very strong association for these two pathways. Other significant pathways of note include the activation of interferon regulatory factors (IRFs) by pattern recognition receptors, retinoic acid-inducible gene I protein (RIG-I)-like receptors in innate immunity, and NF-κB activation by viruses. Figure 2B illustrates the IFN signaling pathway determined by IPA. Notably, in this pathway all of the DM-DE genes are downstream, and none were identified as upstream signaling molecules. IPA also identified 39 upstream regulators (|Z-score| ≥ 2) of the DM-DE genes that showed differential expression between SLE cases and controls in whole blood (Figure 2C). 

**Table 3:** Differential expression of hypomethylated genes in kidney biopsies from independent SLE patients with lupus nephritis.

CpG *	Chr	Pos (bp) ^†^	Gene	Mean Δ*β*	Methylation *p*-Value	Interferon-Regulated ^‡^	Log FC Glomerulus	Log FC Tubulointerstitium
cg16526047	1	949893	*ISG15 ^‖^*	−0.11	1.28 × 10^−4^	IRG	3.32	4.7
cg05696877	1	79088769	*IFI44L ^Δ^*	−0.3	6.60 × 10^−6^	IRG	5.14	5.94
cg01079652	1	79118191	*IFI44 ^‖^*	−0.34	5.34 × 10^−4^	IRG	3.94	4.76
cg17515347	1	159047163	*AIM2*	−0.09	3.01 × 10^−12^	IRG	0.58	NS
cg01028142	2	7004578	*CMPK2 ^Δ^*	−0.33	7.98 × 10^−8^	IRG	NS	NS
cg10959651	2	7018020	*RSAD2*	−0.13	3.14 × 10^−14^	IRG	4.31	3.36
cg14126601	2	37384708	*EIF2AK2 ^Δ^*	−0.1	5.55 × 10^−16^	IRG	1.54	1.72
cg15768138	2	219030752	*CXCR1*	−0.11	7.38 × 10^−27^		0.68	−0.17
cg08122652	3	122281939	*PARP9-DTX3L ^Δ^*	−0.38	1.11 × 10^−9^	IRG	NS	NS
cg06981309	3	146260954	*PLSCR1 ^Δ^*	−0.24	6.41 × 10^−31^	IRG	1.92	2.07
cg02694620	3	172109284	*FNDC3B*	−0.11	3.80 × 10^−3^		NS	0.47
cg15065340	3	195632915	*TNK2 ^‖^*	−0.16	4.04 × 10^−3^		0.38	−0.4
cg07809027	4	15007205	*CPEB2*	−0.1	2.08 × 10^−14^		NS	NS
cg02215171	4	89379156	*HERC5 ^¶^*	−0.09	4.48 × 10^−18^	IRG	3.16	1.96
cg05883128	4	169239131	*DDX60*	−0.25	2.13 × 10^−5^	IRG	1.11	2.31
cg08099136	6	32811251	*PSMB8*	−0.11	1.43 × 10^−4^	IRG	0.76	2.51
cg00052684	6	35694245	*FKBP5 ^§^*	−0.16	1.65 × 10^−3^		−1.27	−2.77
cg05994974	7	139761087	*PARP12 ^‖^*	−0.15	6.89 × 10^−5^	IRG	2.26	1.86
cg14864167	8	66751182	*PDE7A*	−0.35	1.21 × 10^−9^		NS	NS
cg12110437	8	144098888	*LY6E ^◊^*	−0.2	3.14 × 10^−9^	IRG	1.28	1.23
cg03848588	9	32525008	*DDX58 ^◊^*	−0.1	4.34 × 10^−4^	IRG	2.89	2.59
cg06188083	10	91093005	*IFIT3 ^◊^*	−0.25	6.18 × 10^−8^	IRG	2.59	3.14
cg05552874	10	91153143	*IFIT1 ^◊^*	−0.26	6.01 × 10^−16^	IRG	2.24	2.77
cg23570810	11	315102	*IFITM1*	−0.27	1.43 × 10^−18^	IRG	2.24	3.29
cg17990365	11	319718	*IFITM3*	−0.16	8.78 × 10^−295^	IRG	2.24	2
cg08926253	11	614761	*IRF7 ^‖^*	−0.17	2.01 × 10^−9^	IRG	2.8	1
cg08577913	11	4415193	*TRIM21*	−0.1	1.74 × 10^−3^	IRG	1.35	0.77
cg12461141	11	5710654	*TRIM22*	−0.1	6.35 × 10^−25^	IRG	1.73	2.86
cg26811705	11	118781408	*BCL9L*	−0.09	1.64 × 10^−3^		NS	NS
cg19347790	12	81332050	*LIN7A*	−0.09	1.87 × 10^−4^		NS	−0.57
cg25800166	12	113375896	*OAS3*	−0.13	5.36 × 10^−5^	IRG	3.77	1.1
cg19371652	12	113415883	*OAS2*	−0.11	2.24 × 10^−5^	IRG	4.86	1.74
cg03753191	13	43566902	*EPSTI1 ^¶^*	−0.1	9.23 × 10^−5^	IRG	NS	NS
cg00246969	13	99159656	*STK24*	−0.11	6.26 × 10^−6^		NS	0.28
cg07839457	16	57023022	*NLRC5*	−0.23	6.10 × 10^−6^	IRG	NS	NS
cg23571857	17	6658898	*XAF1*	−0.1	1.46 × 10^−8^	IRG	3.14	3.05
cg23378941	17	64361956	*PRKCA*	−0.11	6.89 × 10^−5^	IRG	−0.48	−0.08
cg25178683	17	76976267	*LGALS3BP*	−0.16	2.0 × 10^−8^	IRG	0.57	1.49
cg07573872	19	1126342	*SBNO2*	−0.15	2.77 × 10^−3^	IRG	NS	NS
cg07839313	19	17514600	*BST2 ^‖^*	−0.12	3.48 × 10^−3^	IRG	NS	2.91
cg21549285	21	42799141	*MX1 ^Δ^*	−0.47	6.59 × 10^−13^	IRG	4.05	4.64
cg19460508	22	44422195	*PARVB*	−0.1	1.64 × 10^−3^		0.28	NS
cg20098015	22	50971140	*ODF3B*	−0.21	9.88 × 10^−83^	IRG	NS	NS

Differential gene expression values come from GSE32591: kidney glomerulus and tubulointerstitium WHO class 3/4 lupus nephritis versus control samples. NS indicates not significant FDR *p*-value > 0.2). * CpGs with *p* < 0.01 and Δ*β* < −0.085. ^†^ Positions are from Build 37. ^‡^ As defined by Interferome [37]. ^§^ SLE patients show decreased expression in both kidney tissues. ^‖^ Hypomethylation of this gene at the same CpG site has been reported in SLE patients with renal involvement [12]. ^¶^ Hypomethylation of this gene at a different CpG site has been reported in SLE patients with renal involvement [12]. ^Δ^ Hypomethylation of this gene at the same CpG site has been reported in SLE patients with and without renal involvement [12]. ^◊^ Hypomethylation of this gene at a different CpG site has been reported in SLE patients with renal involvement [12].

**Figure 3:**
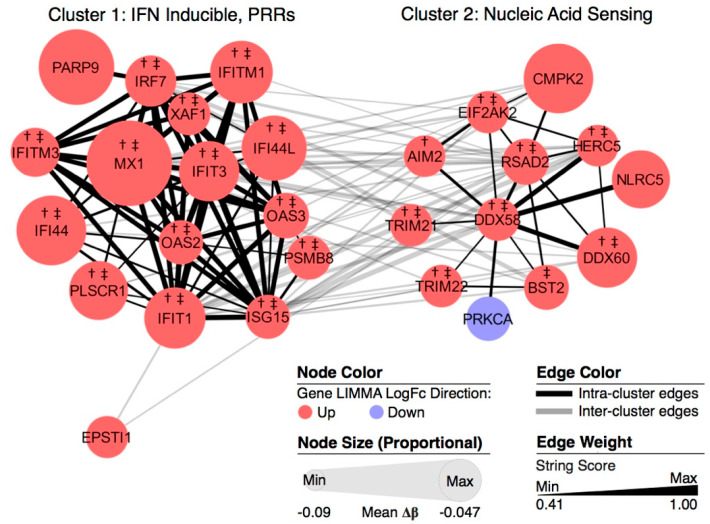
MCODE clustering of hypomethylated genes showing differential expression in independent SLE cohorts. A network scoring degree cutoff of 2, node score cutoff of 0.2, k-Core of 2, and a max depth of 100 were applied. Node color indicates log2(FC) direction and node size is inversely scaled with ∆β (larger nodes are more strongly hypomethylated). Edge weight is scaled by STRING protein–protein connectivity score. All upregulated genes present in clusters were also upregulated in inactive SLE WB samples. †, upregulated in kidney glomerulus, WHO class 3/4. ‡, upregulated in kidney tubulointerstitium, WHO class 3/4.

The DM-DE genes were further analyzed in an additional pathway analysis using the MCODE clustering algorithm and STRING networking scores. Two distinct yet related clusters emerged (Figure 3). As expected, there was an enrichment of genes in the IFN-inducible/pattern recognition receptor pathway. As visually represented by the colors of the nodes and node outlines in Figure 3, all genes in this cluster were upregulated in both active and inactive SLE patients; all of these except *PARP9* were overexpressed in both kidney tissues.

The second cluster was comprised of genes involved in the nucleic acid-sensing pathway, a primary antiviral defense in vertebrates as well as a mechanism to respond to intracellular nucleic acids of cellular origin. There were strong links among the genes in these two clusters as this nucleic acid response of the innate immune system results in the production of type 1 interferon (i.e., INF-α and INF-β) and expression of interferon stimulated genes [59]. These hypomethylated genes showed increased expression in both active and inactive SLE patients; the lone exception observed was the reduced expression of *PRKCA* in active SLE patients. As in the IFN-inducible/pattern recognition receptor pathway, the majority of these nucleic acid-sensing pathway genes were expressed in both kidney tissues. The gene DEAD H-box helicase 58 (*DDX58*), which encodes for retinoic acid-inducible gene I (*RIG-I*) [60], was the central node and exhibited the strongest and most numerous links to other genes within the cluster.

### 3.5. Potential Drug Targets

The DM-DE genes were analyzed for potential gene–drug interactions (Table 4). As evidence of its potential utility, this approach identified methotrexate, a lupus therapy, targeting *EPSTI1*. Twelve of the DM-DE genes are linked to drugs that are currently in ongoing clinical trials, primarily trials related to cancer (Table 4). The drug target analysis also identified 24 additional FDA-approved drugs linked to genes associated with the nucleic acid-sensing or the interferon-inducible pathways. These drugs could merit careful consideration for future clinical trials in SLE.

## 4. Discussion

Environmental challenges coupled with genetic susceptibility are often hypothesized to cause the innate and adaptive immune system to become chronically active, causing failure to recognize subsequent autoimmune disease [61]. Aging and environmental exposures such as smoking, chemicals, diet, and viral pathogens predictably trigger methylation or demethylation at CpG sites. Altered methylation of a CpG site changes the accessibility of transcriptional elements to specific regions, which leads to regulation of gene expression. The relationship between DNA methylation and gene expression is complex, including being influenced by specific tissues/cells [62,63,64]. However, in general, DNA methylation in promoter regions is often inversely correlated with gene expression. The above paradigm is consistent with the results of this multi-omic study, which has demonstrated that genes involved in the nucleic acid-sensing and interferon-inducible pathways were observed to be hypomethylated in SLE-affected MZ twins and upregulated in independent SLE cohorts. Despite the clear biological importance of tissue-specific methylation and gene expression, here, the high concordance of hypomethylated genes in whole blood with increased gene expression across a variety of tissues from multiple independent cohorts suggests a high fidelity of the DNA methylation-gene expression relationship at these loci. 

Every epigenome-wide study of SLE to date, including this one, has identified hypomethylation of multiple type I IFN-related genes. While there is no doubt that stimulation of the type I IFN pathway is important in SLE, the mechanism by which this stimulation occurs will be unique for each SLE patient. Interferon induction occurs due to activation of one of several types of pattern recognition receptors, which are programmed to respond to double-stranded DNA (dsDNA), double-stranded RNA (dsRNA), or single-stranded RNA (ssRNA). The type of nucleic acid (NA) present will depend on the species and cell type producing the NA. Furthermore, the NA may leak into the cytosome where its recognition is again specific to the receptor activated. In our study, bioinformatic analysis identified the NA-sensing pathway, with DEAD/H-Box helicase 58 (*DDX58*) as the central node (Figure 3). *DDX58* encodes for retinoic acid-inducible gene I (*RIG-I*), which recognizes ssRNA. In contrast to Toll-like receptors (TLRs), which recognize NAs in the endosome, RIG-I-like receptors (RLRs) interact with mitochondrial antiviral signaling protein (*MAVS*) in the cytosol [65]. *MAVS* subsequently phosphorylates interferon regulatory factors 3 (*IRF3*) to stimulate type 1 IFN expression. The NA-sensing pathway generated by our analysis also included absent in melanoma 2 (*AIM2*), a cytosolic dsDNA-sensing protein that activates the inflammasome, further emphasizing the plausible role of this pathway in initiating lupus inflammation [66,67].

The cascade of functional consequences resulting from genetic variation and unique environmental exposures will differ for each individual SLE patient. While some SLE patients (10–30%) will present no IFN signature [68], others will overexpress IFN through one of the several mechanisms described above. The DM-SE gene list we prioritized may be a useful tool in grouping SLE patients into DA receptor groups, or “endotypes” as they have been termed by Mustelin et al. [68] Therapies targeting helicases such as *RIG-I*, *MAV*S, or *AIM2* could prove useful for SLE. One such inhibitor of *RIG-I*, enhancer of zeste homolog 2 (*EZH2*), has been shown to play an epigenetic role in SLE and was proposed as a therapeutic target by Tsou et al. [60]. Network analyses and public database queries of our DM-DE genes yielded a list of genes whose products predict gene–drug interactions. The resulting list includes methotrexate, a drug used for the treatment of lupus. The remaining gene–drug interactions we identified merit thorough scrutiny as they could be candidates for future trials. 

Three recent studies have observed aberrant methylation of IFN genes in SLE patients with renal involvement [12,19,22]. A summary of the literature (Additional File 2) shows our study’s consistencies with these published findings. While hypomethylation of these genes has been confirmed in CD4+ T cells and peripheral blood, no SLE study to date has examined genome-wide DNA methylation in kidney biopsies. By considering differential gene expression derived from the micro-dissected glomerulus and tubulointerstitium kidneys in an independent cohort of SLE patients, in conjunction with the significance of aberrant methylation in the MZ twin data, this study corroborates many of the loci previously published as being hypomethylated in lupus nephritis patients. 

The lack of any differentially methylated genes on the X chromosome is noteworthy given the 9:1 female to male gender bias in SLE. This result is not fully explained by the fact that older female MZ twins show a strong tendency for the same X chromosome to be inactivated [69,70] as the lack of differentially methylated sites on the X chromosome in this study is consistent with previous studies of unrelated individuals [11,15,17,18,19,20,21,23,52]. Jeffries et al., using the Illumina Infinium Human Methylation27 array, did observe differential methylation of CpGs in *PCTK1, ARAF, RRAGB*, and *SNX12* on the X chromosome [11], but no studies utilizing the more recent arrays replicate these findings. In our MZ twin study, CpG sites associated with *SNX12* had a minimum *p*-value = 0.02 (change in β = −0.04), but none of the other three genes had *p*-values < 0.05. Thus, to date, methylation patterns among genes located on the X chromosome do not appear to explain a substantial portion of the risk of SLE.

Within this study, the genomic locations of hypomethylated CpG sites were highly skewed toward CpG shores (0–2 kb from island) and shelves (2–4 kb from island) instead of islands. Here, only 5 of 59 CpG sites were in a CpG island, despite nearly one third of the CpG sites on the Illumina HumanMethylation450 BeadChip being in a CpG island (Supplemental Figure S2). Our findings are consistent with those of Yeung et al., who demonstrated that most CpG sites hypomethylated in their lupus patients, when compared to controls, were located in CpG shores [21]. These data corroborate the hypothesis that CpG islands tend to have lower methylation rates than less dense CpG regions (e.g., shores and shelves) and that lower density allows for greater methylation autonomy in response to the environment, leading to increases in potential functional significance of the shores and shelves.

There are several limitations of this multi-omics study. One limitation was the modest sample size, as a larger sample would provide the potential to identify additional differentially methylated regions and pathways. However, it is important to recognize the power and value of a discordant MZ twin study design to reduce confounding based on genetic and environmental background. Further, the modest sample size does not negate the positive findings. There were only three discordant MZ twin pairs in the discovery cohort, but we replicated these results in an independent cohort of four MZ twin pairs. Given the number of samples, we were unable to construct and adjust for the full cell composition of the peripheral blood samples as the limited degrees of freedom precluded the robust use of deconvolution methods. Adjusting for latent methylation components in our analysis, while dampening the associations slightly, still identified the same IFN signature. Further, the collective results are supported by larger, independent case–control studies (described in Additional File 2), and we have shown that our methylation results correlate with gene expression in multiple cell types and tissues in independent SLE case–control studies; many were also identified as eQTMs in a Japanese cohort of 100 healthy individuals. We recognize that our cross-sectional study design (i.e., discovery, replication) cannot separate causality from response to disease, but the consistency of differentially methylated regions with the differentially expressed genes from independent gene expression studies is informative and helps identify epigenetically modified genes and pathways that are important in SLE.

## 5. Conclusions

The intersection of hypomethylated genes from MZ twins and upregulated genes from multiple independent cohorts and cell types were attributed to two distinct but integrated biologic pathways: the nucleic acid-sensing pathway and the IFN-inducing pathway. The source, type, and location of nucleic acids found in an SLE patient determine how and by which receptor the NA is recognized, and ultimately which IRF is stimulated. A multi-omics approach could allow classification of patients into different endotypes and possible treatment groups. Informatically linking the DM-DE genes to drug therapies identified a list of compounds that could be critically evaluated as potential candidates for future trials, either broadly for SLE or for individuals with specific hypomethylation signatures. 

## Figures and Tables

**Table 4:** Predicted drugs targeting hypomethylated genes and associated pathways with ∆β < −0.085.

CpG *	Chr	Pos(bp) ^†^	Gene	Mean ∆β	*p*-Value	STITCH [43]	IPA ^‡^	DGIdb [44]
cg16526047	1	949893	*ISG15*	−0.11	1.28 × 10^−4^			Irinotecan ^F^
cg10959651	2	7018020	*RSAD2*	−0.13	3.14 × 10^−14^	Fludarabine ^F^		
cg14126601	2	37384708	*EIF2AK2*	−0.1	5.55 × 10^−16^			Indirubin derivative E804
cg15768138	2	219030752	*CXCR1*	−0.11	7.38 × 10^−27^	Reparixin ^D^	Reparixin ^D^	SCH-527123, Ketoprofen ^F^
cg06981309	3	146260954	*PLSCR1*	−0.24	6.41 × 10^−31^	Wogonin ^G^		
cg15065340	3	195632915	*TNK2*	−0.16	4.04 × 10^−3^	Dasatinib^−1 F^	Osimertinib ^F^, Vemurafenib^F^	Debromohymenialdisine
cg08099136	6	32811251	*PSMB8*	−0.11	1.43 × 10^−4^	Carfilzomib^4 F^,Oprozomib ^D^, Bortezomib^6 F^	Carfilzomib^4 F^	Carfilzomib^4 F^,
cg00052684	6	35694245	*FKBP5*	−0.16	1.65 × 10^−3^	Rapamycin/Sirolimus^2 F^, Tacrolimus^5 F^		Venlafaxine ^F^, Clomipramine ^F^
cg14864167	8	66751182	*PDE7A*	−0.35	1.21 × 10^−9^			Ketotifen ^F^, Dyphylline ^F^
cg12110437	8	144098888	*LY6E*	−0.2	3.14 × 10^−9^		DLYE5953A^D^	
cg06188083	10	91093005	*IFIT3*	−0.25	6.18 × 10^−8^	Imidazoles ^D^		
cg08926253	11	614761	*IRF7*	−0.17	2.01 × 10^−9^	Hesperidin ^D^		
cg03753191	13	43566902	*EPSTI1*	−0.1	9.23 × 10^−5^	Methotrexate ^F T^, Vinblastine ^F^, Doxorubicin ^F^, Cisplatin ^F^		
cg00246969	13	99159656	*STK24*	−0.11	6.26 × 10^−6^	Staurosporine ^D^		
cg23378941	17	64361956	*PRKCA*	−0.11	6.89 × 10^−5^	Staurosporine ^D^	Aprinocarsen	Midostaurin ^F^, Enzastaurin ^D^, Quercetin ^D G^, Aprinocarsen, Ruboxistaurin ^D^, Ingenol Mebutate ^FW^, Bryostatin ^D^, Sotrastaurin Acetate ^D^, Tamoxifen^2 F^
cg07839313	19	17514600	*BST2*	−0.12	3.48 × 10^−3^	Resveratrol^6 D G^		
cg21549285	21	42799141	*MX1*	−0.47	6.59 × 10^−13^	Mitomycin C ^F^, Colchicine ^F^		
cg19460508	22	44422195	*PARVB*	−0.1	1.64 × 10^−3^	Lovastatin^3 F^		Bortezomib^6 F^

* CpGs with *p* < 1 × 10^−3^ and Δ*β* < −0.085. ^†^ Positions are from Build 37. ^‡^ Qiagen Bioinformatics: ingenuity.com ^F^ FDA approved. ^D^ Ongoing clinical trial or DiD ^G^ GRAS. ^T^ Known utility in lupus therapy. ^FW^ Ingenol mebutate is FDA-approved in the US but withdrawn in the EU. Numbers in superscript are CoLTS scores and range from −16 to +11.

## Data Availability

All gene expression datasets are publicly available from the Gene Expression Omnibus (GEO) (Supplemental Table S1). The methylation data from the three monozygotic twins are available upon request from the authors. Samples, including DNA, are available from the Lupus Family Registry and Repository.

## Supplementary Materials

The following are available online at https://www.mdpi.com/article/10.3390/genes12121898/s1: Table S1: GEO repository datasets interrogated for differential expression between SLE cases and controls; Table S2: Clinical characteristics of monozygotic twins discordant for SLE; Table S3: Differential expression of hypomethylated genes in peripheral cell types from independent SLE cohorts; Table S4: Differentially methylated probes from three monozygotic twin pairs discordant for SLE; Figure S1: Area under the receiver operating characteristic curve (AUC) calculated at regular intervals between 0 and −0.15 in four cell types; Figure S2: Proportions of significantly associated CpGs (as defined in Table 1) located in islands, shores, shelves, and other content categories; Additional File 1: Differential methylation in MZ twins discordant for SLE; Additional File 2: Replication of CpG sites in Table 1 across published epigenome-wide SLE studies.
