# Supplementary material for: Nucleic Acid-Sensing and Interferon-Inducible Pathways Show Differential Methylation in MZ Twins Discordant for Lupus and Overexpression in Independent Lupus Samples: Implications for Pathogenic Mechanism and Drug Targeting
Source: Genes (Basel). 2021 Nov 26;12(12):1898. doi: 10.3390/genes12121898 (PMC8701117; doi:10.3390/genes12121898)
Supplement: Supplementary file 1 [file genes-12-01898-s001.zip › Additional_File_1.pdf]

Methylation results

LFRR 3 MZ twin pairs | Danish 4 MZ twin pairs - CD14 n

cpg	gene	chr	pos	pvalue_whole_blood	mean_db_cd14	x2_cd14
cg21549285	MX1	21	41721011	$6.59x10^{-13}$	-0.237801773	5.065927204
cg08122652	PARP9	3	123764629	$1.11x10^{-9}$	-0.276622975	6.937094881
cg22930808	PARP9	3	123764571	$6.74x10^{-126}$	-0.26939105	5.937561678
cg14864167	PDE7A	8	66913736	$1.21x10^{-9}$	-0.192931125	6.140928344
cg00959259	PARP9	3	123764665	$1.32x10^{-56}$	-0.1488192	7.385325467
cg01028142	CMPK2	2	6922029	$7.98x10^{-8}$	-0.27166395	9.722499767
cg22862003	MX1	21	41719458	$1.62x10^{-25}$	-0.210177413	7.846312629
cg13304609	IFI44L	1	78857750	$1.58x10^{-14}$	-0.246675675	5.30053783
cg23570810	IFITM1	11	305102	$1.43x10^{-18}$	-0.165794625	10.86332976
cg03607951	IFI44L	1	78858174	$7.23x10^{-22}$	-0.08808745	2.029064517
cg05552874	IFIT1	10	91143123	$6.01x10^{-16}$	-0.190213455	9.045510785
cg06188083	IFIT3	10	91082985	$6.18x10^{-8}$	-0.15741695	6.269685706
cg03038262	IFITM1	11	305262	$4.41x10^{-40}$	-0.20111875	7.977444098
cg06981309	PLSCR1	3	147743644	$6.41x10^{-31}$	-0.11756405	3.48750165
cg06872964	IFI44L	1	78857838	$1.05x10^{-71}$	-0.143949025	7.91227882
cg05523603		22	49319967	$5.51x10^{-14}$	-0.120780625	14.88942213
cg26312951	MX1	21	41719717	$6.28x10^{-15}$	-0.082715263	6.443949294
cg20098015	ODF3B	22	49318006	$9.88x10^{-83}$	-0.06408665	4.622023698
cg12110437	LY6E	8	144170263	$3.14x10^{-9}$	-0.141687125	3.13569181
cg20045320		11	309555	$4.85x10^{-17}$	-0.136491275	14.9990855
cg08926253	IRF7	11	604761	$2.01x10^{-9}$	-0.1751758	16.10348337
cg17990365	IFITM3	11	309718	$8.78x10^{-295}$	-0.1835469	10.97677432
cg25178683	LGALS3BP	17	74487862	$2.01x10^{-8}$	-0.12635275	17.41380992
cg04927537	LGALS3BP	17	74487686	$2.77x10^{-10}$	-0.1070397	33.66429537
cg22708150	LY6G5C	6	31757598	$1.05x10^{-19}$	-0.006755675	0.050335875
cg10552523	IFITM1	11	303478	$5.90x10^{-115}$	-0.1160107	9.665841682
cg10959651	RSAD2	2	6935471	$3.14x10^{-14}$	-0.07780988	4.089153838
cg24740632		5	134514577	$2.26x10^{-60}$	-0.0086746	0.451470743
cg12013713	PARP12	7	139407140	$1.44x10^{-16}$	-0.0766746	5.769553046
cg06012695		6	28878572	$3.59x10^{-16}$	-0.034712667	1.208676684
cg16785077	MX1	21	41713737	$8.45x10^{-27}$	-0.0866469	6.698642327
cg02314339		10	91010633	$1.72x10^{-08}$	-0.059648775	28.9356976
cg21873524		4	191179738	$1.03x10^{-55}$	0.00783375	0.175059717
cg15768138	CXCR1	2	218738997	$7.38x10^{-27}$	-0.006321867	0.018581865
cg20566897	IFITM1	11	303527	$7.00x10^{-62}$	-0.09257945	22.29067256
cg23571857	XAF1	17	6599622	$1.46x10^{-8}$	-0.0564352	15.42253535
cg12461141	TRIM22	11	5667230	$6.35x10^{-25}$	-0.0094358	0.400056297
cg14126601	EIF2AK2	2	37238212	$5.55x10^{-16}$	-0.076897825	4.404725433
cg26337070	ATOH8	2	85853384	$7.55x10^{-9}$	-0.058026125	0.858495042
cg07809027	CPEB2	4	14616303	$2.08x10^{-14}$	-0.0803636	30.53271108

cg16503797		18	17730803	$5.39x10^{-12}$	0.01924085	0.50891441
cg20190772	KIAA0146	8	48735049	$1.40x10^{-8}$	-0.070328175	12.05066769
cg04781494	CASP10	2	201755491	$8.39x10^{-8}$	-0.00396995	0.024149583
cg17786255	SGMS2	4	109033838	$2.01x10^{-16}$	0.0121885	0.11470528
cg02215171	HERC5	4	89598179	$4.48x10^{-18}$	-0.0490381	1.365298518
cg02247863		22	49330281	$2.51x10^{-13}$	-0.078810425	9.983164434
cg17555806		10	74118123	$1.51x10^{-8}$	0.022079625	0.720329252
cg13411554	CACNA1D	3	53675316	$8.66x10^{-8}$	-0.085056675	4.580706282
cg02556393	MECOM	3	170349399	$3.14x10^{-95}$	-0.018857125	0.487313246
cg17515347	AIM2	1	157313787	$3.01x10^{-12}$	-0.01679485	0.223388789
cg10549986	RSAD2	2	6935604	$1.95x10^{-91}$	-0.04798899	1.650936537
cg25138053		6	31475995	$3.67x10^{-15}$	-0.002368925	0.005920041
cg06102678		8	81653883	$1.00x10^{-8}$	-0.0132492	0.435609351
cg08272268	ZNF281	1	198646682	$4.33x10^{-15}$	-0.002783375	0.00305944
cg14910175		10	131730944	$1.56x10^{-11}$	-0.00056225	0.000341345
cg15871086		18	54677575	$2.08x10^{-11}$	-0.0315256	13.98419823
cg05543864	GGT1	22	23309755	$1.44x10^{-45}$	-0.0503681	4.067536366
cg07292773		6	156759869	$2.22x10^{-17}$	-0.00276405	0.012180545
cg23352030	PRIC285	20	61668913	$2.36x10^{-11}$	0.000315475	8.84931E-05

Methylation results for CpG sites listed in Table 1.

mean_db = mean delta beta (i.e. mean difference in methylation beta between affected and unaf

x2 = chi squared value

Geen highlighting indicates that the direction of delta beta is opposite in LFRR and Danish twin co

Yellow highlighting indicates that the p-value for differential methylation was less that 0.05 in the

ıonocytes	Danish 4 MZ twin pairs - CD4 T cells			Danish 4 MZ twin pairs - CD 19 B	
**pvalue_cd14**	**mean_db_cd4t**	**x2_cd4t**	**pvalue_cd4t**	**mean_db_cd19b**	**x2_cd19b**
0.024400649	-0.194845443	4.738057445	0.029502366	-0.25074648	8.263077549
0.008442612	-0.197376675	5.132678472	0.023479468	-0.217248425	5.134961813
0.01482151	-0.205405375	5.732973546	0.016649268	-0.181173425	30.80982426
0.013208795	0.022013925	0.497226751	0.480721205	-0.1591725	4.0679579
0.006575817	-0.136082	5.669586942	0.01726152	-0.027929867	0.211607608
0.001820258	-0.2729289	4.275990526	0.038654428	-0.24963915	7.828894953
0.005092447	-0.182722275	3.987071647	0.045850685	-0.195596675	7.696520595
0.021318841	-0.11128025	4.813579019	0.028236343	-0.1438988	3.894596576
0.000980872	-0.03059629	6.802227904	0.00910442	-0.18519025	4.256688821
0.154315567	-0.23761175	6.442278097	0.011143565	-0.15645795	5.954201821
0.002633406	-0.2253475	3.3332366	0.067893147	-0.270465325	7.752488277
0.012282093	-0.0678028	2.315593909	0.128082372	-0.061100725	2.057768361
0.004736377	-0.00830156	2.389593043	0.122145422	-0.2126947	5.969773105
0.061833836	-0.200140825	5.05462535	0.024560292	-0.2095402	6.960909296
0.004910038	-0.1734456	6.030395903	0.014061579	-0.1901231	6.645264584
0.000114	-0.08011005	13.45744462	0.000244036	0.00159105	0.000718577
0.011133087	-0.044662725	3.487440505	0.061836121	-0.10270557	11.82947137
0.031563978	-0.0916362	4.35162318	0.036973691	-0.11515885	8.096528083
0.076595884	-0.044071767	0.959100766	0.327413543	-0.017881833	0.577974209
0.000107563	-0.046484325	1.707146328	0.191356039	-0.149563225	8.037352258
5.99736E-05	-0.114694575	2.268487917	0.132028664	-0.09392395	3.860113339
0.000922609	-0.060545175	3.140808679	0.076355941	-0.149219925	6.441638294
3.00634E-05	-0.06464835	5.019332228	0.025065834	-0.09679205	8.427377235
6.55E-09	-0.063386175	6.116582294	1.34E-02	-0.102061075	9.836064903
0.822479854	-0.02237835	2.753460586	0.097044183	0.008212475	0.068645684
0.001877257	-0.016729627	1.908900282	0.167085468	-0.011217778	1.14986621
0.043159244	-0.049896338	7.240123214	0.007129209	-0.123464725	4.444456777
0.501637348	-0.08065255	2.591223841	0.107457276	-0.046803525	3.44506564
0.016306181	-0.08957235	2.959166796	0.085391934	-0.084207375	8.436194868
0.27159436	0.010259667	0.105525263	0.745297233	-0.018852	0.426507136
0.009648635	-0.06593405	5.961836117	0.014618809	-0.0092719	0.634839031
7.48E-08	-0.0406004	3.475424727	6.23E-02	-0.0153611	7.543674632
0.675653676	0.035746867	7.368195349	0.006638753	-0.03380975	0.7770055
0.891572019	-0.01496815	0.278128222	0.597930679	0.045984	3.837131926
2.34E-06	-0.020313375	1.26333653	2.61E-01	-0.03683099	6.485108099
8.59571E-05	-0.080080525	5.982627503	0.014447463	-0.07549215	5.579031245
0.527060184	-0.08292925	5.811033706	0.01592593	0.013654575	0.127638473
0.035839494	-0.1356826	8.522439295	0.00350794	-0.08735905	4.147728856
0.354160186	-0.0203052	0.74111328	0.389304488	-0.0163249	0.205618544
3.28E-08	-0.05430595	1.37431619	2.41E-01	-0.033639325	1.295151741

0.475609178	-0.03618365	4.461877347	0.034659386	-0.033396	2.483831168
0.000517738	-0.042823375	0.69244092	0.405335372	-0.084886475	27.93176023
0.876504911	-0.04636825	2.957696667	0.085469615	-0.013916375	1.510054765
0.734849543	-0.034586275	13.65378623	0.000219798	-0.017285575	0.451071517
0.242621309	-0.137347375	4.147776935	0.041689223	-0.093326675	7.007872034
0.00157978	-0.0488933	3.456217582	0.063014308	-0.01890305	7.184063905
0.39603593	0.0013083	0.004278403	0.947847956	0.048101825	1.081889322
0.032333885	-0.067077225	2.093867202	0.147891291	-0.063354725	3.245886419
0.485128258	-0.062989075	2.706597656	0.099933923	-0.063257625	5.102479687
0.636469874	0.022808975	0.206864819	0.649235904	0.064133125	2.585899246
0.198831511	-0.02819274	2.074142735	0.149814094	-0.162851855	2.406362386
0.938669842	-0.03239772	0.07358393	0.786188316	0.002317725	0.015835174
0.509249284	-0.040992175	5.1659119	0.023034362	-0.0179709	0.310739118
0.955889737	-0.019494825	0.669965084	0.413063781	-0.014963875	0.435270416
0.985259511	-0.008572375	0.079770233	0.777609026	-0.0217718	1.598582257
0.000184354	-0.01094555	0.226351766	0.634242234	-0.03948685	74.47048799
0.043714988	-0.0466726	4.13622739	0.041974618	-0.030927375	9.776053422
0.912119509	0.030693175	2.214584968	0.136711823	-0.00400905	0.013852491
0.992494349	-0.0062394	0.160614335	0.688591542	0.010468375	0.203637534

[illegible]fected twins)

[illegible]horts.
[illegible] Danish MZ twins cohort.

cells	Danish 4 MZ twin pairs - granulocytes		
**pvalue_cd19b**	**mean_db_granulo**	**x2_granulo**	**pvalue_granulo**
0.004045948	-0.311716378	7.475558337	0.006254217
0.023448603	-0.263188375	6.867272058	0.008778856
2.84593E-08	-0.1943683	6.618986229	0.010089725
0.04370408	-0.197121475	5.432536612	0.01976495
0.645510285	-0.112989075	2.090893845	0.148179351
0.005141753	-0.2372076	4.558675693	0.03275239
0.005532738	-0.21158705	5.456681303	0.019493637
0.048441672	-0.208947	5.832034494	0.015736914
0.039096057	-0.20181265	8.106875791	0.004409766
0.014682249	-0.1038534	3.519880222	0.06063683
0.005363859	-0.228746875	9.453372412	0.002107612
0.151431805	-0.174806375	4.338237342	0.037265485
0.014553153	-0.16779665	4.806688132	0.028349479
0.008330968	-0.1195076	16.77973506	4.19792E-05
0.009941971	-0.122601625	6.047781512	0.01392378
0.978614234	-0.15226555	8.811307825	0.002993694
0.000583005	-0.12039105	2.985961416	0.083989396
0.004435013	-0.1202952	5.963512578	0.014604916
0.447107463	-0.161550225	5.103615344	0.023876029
0.004582247	-0.1419082	10.97147882	0.000925249
0.049446988	-0.1626318	5.821245125	0.015833732
0.011147579	-0.1533688	8.544682805	0.00346533
0.003696131	-0.104662225	3.973397899	0.046224406
1.71E-03	-0.1461688	9.384429597	2.19E-03
0.793318965	-0.00876475	0.335696231	0.562324036
0.283577158	-0.07009785	4.328246496	0.037484843
0.035014728	-0.088317955	3.693947209	0.054610236
0.063440902	0.00141045	0.00033914	0.985307184
0.003678251	-0.104426675	6.073954477	0.013718948
0.51370784	0.0091623	0.045910293	0.83033895
0.425585862	-0.085418175	3.285987185	0.069873597
6.02E-03	-0.083138325	5.133788738	2.35E-02
0.378058532	-0.0282619	324.6222544	0
0.050129202	0.043561825	0.233487179	0.628950107
1.09E-02	-0.07201105	7.571134666	5.93E-03
0.018176775	-0.0528109	6.229334784	0.012565093
0.720893227	-0.058292025	0.992625107	0.319101621
0.041690406	-0.053989475	9.573827229	0.001973709
0.650223436	-0.059334675	1.79353028	0.180496625
2.55E-01	-0.043437575	0.726206491	3.94E-01

0.11502177	-0.039684275	52.64427827	3.99791E-13
1.2567E-07	-0.0701246	32.89054985	9.74961E-09
0.21913073	0.025539	1.205438447	0.272237411
0.501826547	0.0098448	0.113778103	0.735883148
0.008115208	-0.083512125	9.449009591	0.002112632
0.007355392	-0.05941965	2.267622046	0.13210246
0.298275286	-0.047493175	7.201677311	0.007283547
0.071602949	-0.084313575	4.351436902	0.036977735
0.023891665	-0.03591845	2.772729494	0.095882536
0.10781916	-0.053405025	2.935482492	0.086652769
0.120842879	-0.03451995	2.617641936	0.105681384
0.89986025	-0.0223373	1.237777311	0.265899851
0.577226987	-0.0141281	0.453373747	0.500737155
0.509414102	-0.0131987	0.284218438	0.593949539
0.206104241	-0.005413725	0.140898728	0.707389185
0	-0.010307	0.507972581	0.47601783
0.001767994	-0.053874075	3.034288159	0.0815223
0.906308057	0.016643225	1.314734007	0.251539323
0.651800616	0.044282325	1.402399074	0.2363223
