# Supplementary material for: Nucleic Acid-Sensing and Interferon-Inducible Pathways Show Differential Methylation in MZ Twins Discordant for Lupus and Overexpression in Independent Lupus Samples: Implications for Pathogenic Mechanism and Drug Targeting
Source: Genes (Basel). 2021 Nov 26;12(12):1898. doi: 10.3390/genes12121898 (PMC8701117; doi:10.3390/genes12121898)
Supplement: Supplementary file 1 [file genes-12-01898-s001.zip › Additional_File_2.pdf]

CpG	Chr	Pos	Gene
cg13304609	1	79085162	IFI44L
cg06872964	1	79085250	IFI44L
cg03607951	1	79085586	IFI44L
cg01028142	2	7004578	CMPK2
cg10959651	2	7018020	RSAD2
cg10549986	2	7018153	RSAD2
cg14126601	2	37384708	EIF2AK2
cg13411554	3	53700276	CACNA1D
cg22930808	3	122281881	PARP9 -DTX3L
cg08122652	3	122281939	PARP9 -DTX3L
cg00959259	3	122281975	PARP9 -DTX3L
cg06981309	3	146260954	PLSCR1
cg02556393	3	168866705	MECOM
cg07809027	4	15007205	CPEB2
cg02215171	4	89379156	HERC5
cg21873524	4	190942744	
cg25138053	6	31368016	
cg22708150	6	31649619	LY6G5C
cg12013713	7	139760671	PARP12
cg14864167	8	66751182	PDE7A
cg12110437	8	144098888	LY6E
cg06188083	10	91093005	IFIT3
cg05552874	10	91153143	IFIT1
cg10552523	11	313478	IFITM1
cg20566897	11	313527	IFITM1
cg23570810	11	315102	IFITM1
cg03038262	11	315262	IFITM1
cg20045320	11	319555	
cg17990365	11	319718	IFITM3
cg08926253	11	614761	IRF7

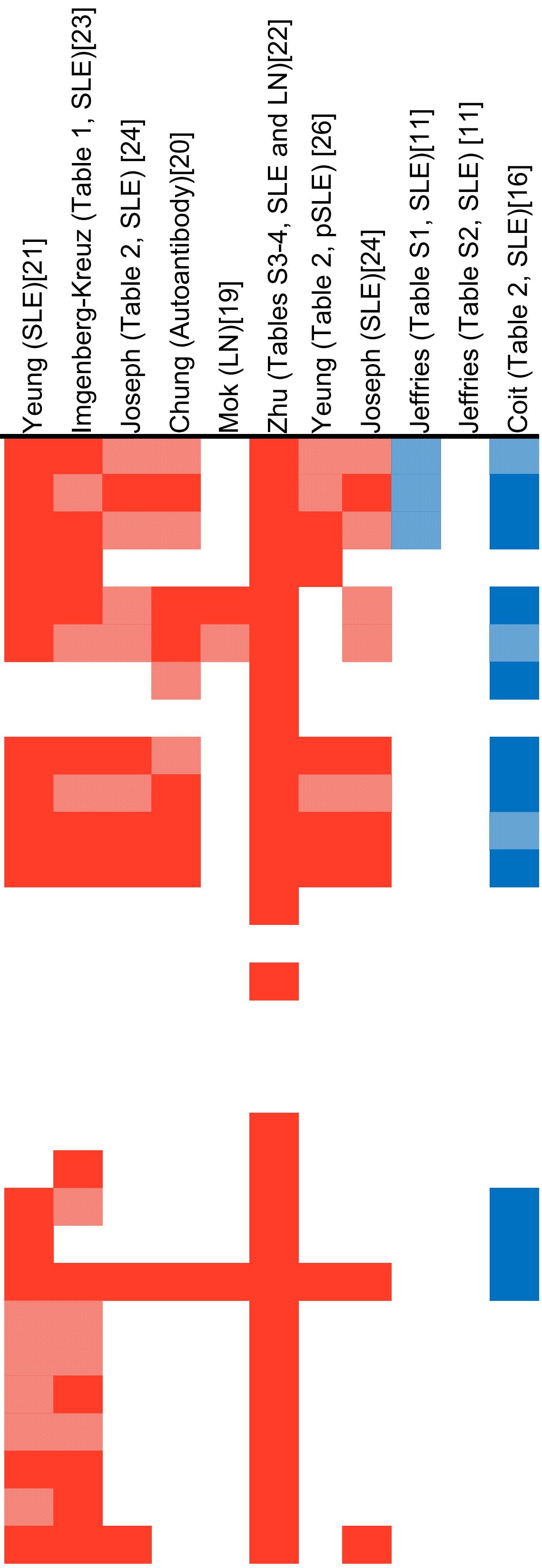

cg12461141	11	5710654	TRIM22
cg04927537	17	76976091	LGALS3BP
cg25178683	17	76976267	LGALS3BP
cg23352030	20	62198469	PRIC285
cg16785077	21	42791867	MX1
cg22862003	21	42797588	MX1
cg26312951	21	42797847	MX1
cg21549285	21	42799141	MX1
cg05543864	22	24979755	GGT1
cg20098015	22	50971140	ODF3B
cg05523603	22	50973101	
cg02247863	22	50983415	
cg17515347	1	159047163	AIM2
cg08272268	1	200380059	ZNF281
cg26337070	2	85999873	ATOH8
cg04781494	2	202047246	CASP10
cg15768138	2	219030752	CXCR1
cg17786255	4	108814389	SGMS2
cg24740632	5	134486678	
cg06012695	6	28770593	
cg07292773	6	156718177	
cg20190772	8	48572496	KIAA0146
cg06102678	8	81491328	
cg17555806	10	74448117	
cg02314339	10	91020653	
cg14910175	10	131840954	
cg23571857	17	6658898	XAF1
cg16503797	18	19476805	
cg15871086	18	56526595	

Replication of CpG sites in Table 1 across published epigenomewide SLE studies to date (July 2
Red indicates methylation was quantified in whole blood or peripheral blood cells.
Blue indicates methylation was quantified in CD4+ T cells.
Purple indicates methylation was quantified in CD19+ B cells.
Yellow indicates methylation was quantified in CD14+ monocytes.
Black indicates methylation was quantified in at least two of CD4+ T cells, CD19+ B cells and CD14+ m
Aqua indicates methylation was quantified in granulocytes.
Green indicates methylation was quantified in neutrophils.
Diagonal stripes indicate that the gene reported is the same, but the CpG site is different.
SLE - Methylation patterns were assessed in SLE patients compared to controls.
pSLE - Methylation patterns were assessed in pediatric SLE patients compared to controls.
LN - Methylation patterns were assessed in SLE patients with nephritis compared to SLE patients witho
Malar - Methylation patterns were assessed in SLE patients with malar rash compared to healthy contrc

Discoid - Methylation patterns were assessed in SLE patients with discoid rash compared to healthy co[illegible]
Autoantibody - Methylation patterns were assessed in SLE patients with anti-dnDNA, anti-SSA, anti-SS[illegible]

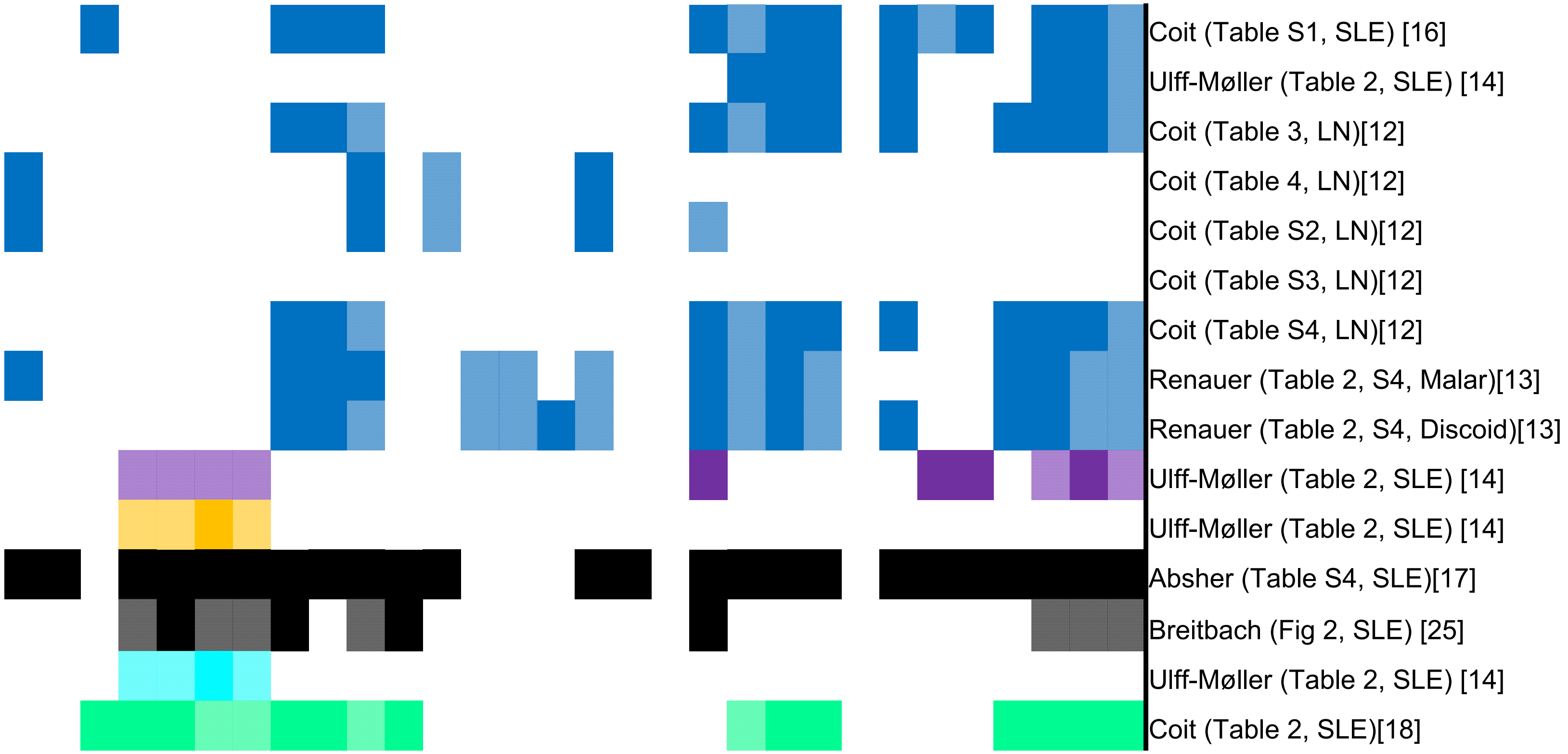
Coit (Table S1, SLE) [16]
Ulff-Møller (Table 2, SLE) [14]
Coit (Table 3, LN)[12]
Coit (Table 4, LN)[12]
Coit (Table S2, LN)[12]
Coit (Table S3, LN)[12]
Coit (Table S4, LN)[12]
Renauer (Table 2, S4, Malar)[13]
Renauer (Table 2, S4, Discoid)[13]
Ulff-Møller (Table 2, SLE) [14]
Ulff-Møller (Table 2, SLE) [14]
Absher (Table S4, SLE)[17]
Breitbach (Fig 2, SLE) [25]
Ulff-Møller (Table 2, SLE) [14]
Coit (Table 2, SLE)[18]

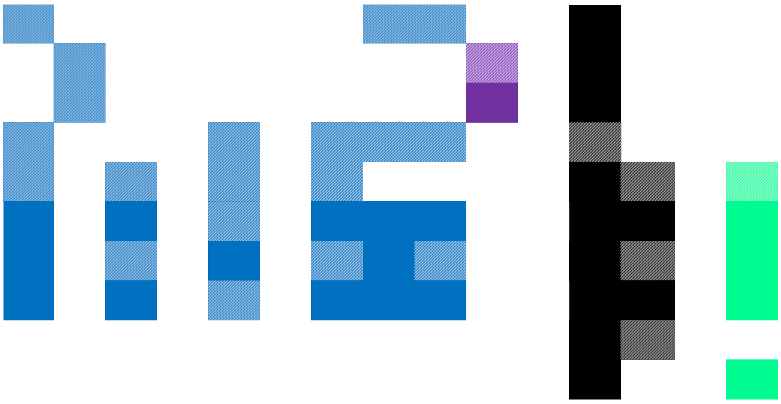

021)

onocytes.

ut nephritis, or healthy controls.
ls.

ntrols.
B, anti-SM, and anti-RNP antibodies compared to SLE patients without antibodies.
