# Supplementary material for: Nucleic Acid-Sensing and Interferon-Inducible Pathways Show Differential Methylation in MZ Twins Discordant for Lupus and Overexpression in Independent Lupus Samples: Implications for Pathogenic Mechanism and Drug Targeting
Source: Genes (Basel). 2021 Nov 26;12(12):1898. doi: 10.3390/genes12121898 (PMC8701117; doi:10.3390/genes12121898)

Table S1. GEO repository datasets interrogated for differential expression between SLE cases and controls.

Dataset	Tissue	SLE Cases	Controls
GSE10325	CD19+ B cells	15	9
GSE4588	CD20+ B cells	7	9
GSE10325	CD4+ T cells	12	9
GSE51997	CD4+ T cells	6	8
GSE38351	CD14+ Monocytes	14	12
GSE39088	Whole Blood	21	64
GSE49454	Whole Blood	157	20
GSE32591	Glomerulus	22*	15
GSE32591	Tubulointerstitium	22*	14

*Berthier Kidney Glomerulus and Tubulointerstitium WHO class 3/4 lupus nephritis patients

Table S2. Clinical characteristics of monozygotic twins discordant for SLE

Clinical Characteristic	Twin Pair 1		Twin Pair 2		Twin Pair 3	
Age at Collection	49		47		67	
SLE	Affected	Unaffected	Affected	Unaffected	Affected	Unaffected
ANA Titer	3240	40	360	40	3240	NEG
ANA Pattern	Nuclear Speckled	Nuclear Speckled	Nucleolar	Nuclear Speckled	Nuclear Homogeneous	None
Anti-dsDNA Titer	NEG	NEG	10	NEG	270	NEG
Anti-Sm	POS	NEG	NEG	NEG	NEG	NEG
Anti-nRNP	POS	NEG	NEG	NEG	NEG	NEG
Anti-Ro	POS	NEG	NEG	NEG	POS	NEG
Anti-La	NEG	NEG	NEG	NEG	POS	NEG
Malar Rash	NEG		UNK		POS	
Discoid Rash	POS		NEG		NEG	
Photosensitivity	NEG		POS		POS	
Oral Ulcers	NEG		NEG		POS	
Arthritis	POS		UNK		POS	
Serositis	NEG		NEG		NEG	
Renal Disorder	NEG		POS		NEG	
Neurologic Disorder	NEG		NEG		NEG	
Hemotologic Disorder	NEG		POS		NEG	

Table S3. Differential expression of hypomethylated genes in peripheral cell types from independent SLE cohorts

						CD14		CD19/20		CD4	
						Active ‡	Inactive ‡	Active‡		Active ‡	Inactive ‡
CpG*	Chr	Pos (bp)†	Gene	Mean $\Delta\beta$	Methylation P-value	logFC	logFC	logFC	logFC	logFC	logFC
cg21549285	21	42799141	MX1	-0.4717	6.59x10^{-13}	2.33	2.2	2.5	2.8	2.95	4.05
cg01079652	1	79118191	IFI44	-0.3352	5.34x10^{-4}	2.35	2.26	5.37	3.67	2.83	3.5
cg05696877	1	79088769	IFI44L	-0.2955	6.60x10^{-6}	4.05	3.82	6.28	5.05	4.59	5.16
cg23570810	11	315102	IFITM1	-0.2736	1.43x10^{-18}	3.51	3.1	2.38	2.2	0.53	0.66
cg05552874	10	91153143	IFIT1	-0.2616	6.01x10^{-16}	3.79	3.37	4.63	4.64	3.28	5.16
cg06188083	10	91093005	IFIT3	-0.2529	6.18x10^{-8}	2.78	2.82	3.87	3.49	4.08	5.5
cg05883128	4	169239131	DDX60	-0.2516	2.13x10^{-5}	1.97	1.73	1.4	2.57	1.67	2.42
cg12110437	8	144098888	LY6E	-0.1991	3.14x10^{-9}	2.17	2.01	1.5	1.02	NS	NS
cg08926253	11	614761	IRF7	-0.1736	2.01x10^{-9}	1.42	1.68	1.72	1.79	1.93	3.03
cg17990365	11	319718	IFITM3	-0.1557	8.78x10^{-295}	1.65	1.5	1.03	1.36	NS	NS
cg25178683	17	76976267	LGALS3BP	-0.1555	2.01x10^{-8}	4.49	4.18	2.12	3.03	1.51	2.51
cg05994974	7	139761087	PARP12	-0.1509	6.89x10^{-5}	1.08	1.42	NS	NS	1.13	1.39
cg10959651	2	7018020	RSAD2	-0.1302	3.14x10^{-14}	4.42	4.25	4.79	3.98	5.09	5.08
cg25800166	12	113375896	OAS3	-0.126	5.36x10^{-5}	2.12	1.97	3	3.29	2.03	3.7
cg07839313	19	17514600	BST2	-0.1192	3.48x10^{-3}	0.5	0.64	1.41	1.32	NS	NS
cg23367341	5	171504064	STK10	-0.1152	3.55x10^{-5}	NS	NS	-0.5	-0.24	NS	NS
cg19371652	12	113415883	OAS2	-0.1147	2.24x10^{-5}	1.95	1.58	0.68	2.07	NS	NS
cg15338782	15	26035581	ATP10A	-0.1135	1.55x10^{-5}	1.89	1.73	NS	NS	NS	NS
cg16526047	1	949893	ISG15	-0.1134	1.28x10^{-4}	2.73	2.55	3.79	3.33	2.99	3.6
cg04410989	20	35578437	SAMHD1	-0.1104	1.23x10^{-7}	NS	NS	1.56	0.97	NS	NS
cg02694620	3	172109284	FNDC3B	-0.1054	3.80x10^{-3}	1.46	1.74	2.91	1.09	NS	NS
cg23571857	17	6658898	XAF1	-0.1016	1.46x10^{-8}	0.76	0.63	2.01	2.74	1.81	2.69
cg03848588	9	32525008	DDX58	-0.1004	4.34x10^{-4}	2.42	2.34	NS	NS	NS	NS
cg12461141	11	5710654	TRIM22	-0.0998	6.35x10^{-25}	0.76	0.63	NS	NS	NS	NS
cg25954539	6	31323677	HLA-B	-0.0994	4.38x10^{-5}	0.14	0.1	NS	NS	NS	NS
cg26644674	10	3138505	PFKP	-0.0972	7.14x10^{-4}	0.49	1.03	NS	NS	0.91	0.5

cg08577913	11	4415193	TRIM21	-0.0972	$1.74x10^{-3}$	NS	NS	NS	NS	0.71	1.39
cg14126601	2	37384708	EIF2AK2	-0.097	$5.55x10^{-16}$	2.19	1.58	2.57	2.18	2.39	2.26
cg19460508	22	44422195	PARVB	-0.095	$1.64x10^{-3}$	-0.78	-0.53	NS	NS	NS	NS
cg04781494	2	202047246	CASP10	-0.0926	$8.39x10^{-8}$	NS	NS	1.02	1.11	NS	NS
cg02215171	4	89379156	HERC5	-0.0923	$4.48x10^{-18}$	2.78	2.55	3.22	3.41	2.72	3.02
cg17515347	1	159047163	AIM2	-0.0904	$3.01x10^{-12}$	NS	NS	NS	NS	1.97	1.01
cg10152449	7	2444534	CHST12	-0.0896	$2.64x10^{-3}$	NS	NS	1.95	0.76	NS	NS
cg06112967	2	231085725	SP110	-0.0812	$5.09x10^{-5}$	1.12	1.11	NS	NS	1.04	1.93
cg00598235	4	17580680	LAP3	-0.0811	$3.82x10^{-4}$	0.91	1.03	2.56	2.33	1.62	1.68
cg00159243	12	109023799	SELPLG	-0.0811	$5.10x10^{-4}$	-0.35	-0.24	NS	NS	NS	NS
cg02902617	14	93031178	RIN3	-0.0801	$1.22x10^{-4}$	NS	NS	-0.39	-0.5	NS	NS
cg20842915	7	39665132	RALA	-0.0799	$3.75x10^{-5}$	NS	NS	1.5	0.86	NS	NS
cg16549027	6	43149629	CUL9	-0.0791	$5.26x10^{-6}$	NS	NS	-0.79	-0.5	NS	NS
cg01636591	17	32646156	CCL8	-0.0772	$1.73x10^{-6}$	1.72	1.92	NS	NS	NS	NS
cg11132204	11	9405884	IPO7	-0.0763	$1.22x10^{-4}$	-0.41	-0.39	NS	NS	NS	NS
cg04953958	7	149558544	ZNF862	-0.0758	$8.36x10^{-4}$	NS	NS	-0.68	-0.45	NS	NS
cg12968598	6	47444699	CD2AP	-0.0737	$2.99x10^{-4}$	0.87	1.13	0.46	0.88	NS	NS
cg19107595	7	94285642	PEG10	-0.0707	$4.32x10^{-4}$	NS	NS	-0.24	-1.16	NS	NS
cg24502904	12	53399544	EIF4B	-0.0705	$8.39x10^{-4}$	-1.29	-0.93	-0.49	-1.27	NS	NS
cg12942320	6	44354771	CDC5L	-0.0704	$9.42x10^{-5}$	0.29	0.42	NS	NS	NS	NS
cg13648715	4	77133417	SCARB2	-0.0681	$8.31x10^{-5}$	1.55	2.15	NS	NS	NS	NS
cg03507262	14	95784787	CLMN	-0.0677	$2.39x10^{-3}$	-1.5	-0.65	-1.88	-1.51	NS	NS
cg22177286	2	231728967	ITM2C	-0.0672	$2.11x10^{-3}$	NS	NS	NS	NS	-0.81	-0.59
cg24702826	6	86171768	NT5E	-0.0669	$6.69x10^{-4}$	NS	NS	-1.61	-0.96	NS	NS
cg05949660	6	109777642	MICAL1	-0.0655	$3.53x10^{-9}$	-0.2	-0.28	NS	NS	NS	NS
cg02452732	17	41158611	IFI35	-0.0647	$1.43x10^{-5}$	1.34	1.44	2.3	1.71	1.57	2.76
cg09168222	4	89299733	HERC6	-0.0639	$2.13x10^{-3}$	3.09	3.09	2.76	2.84	2.09	2.87
cg25599012	19	39827384	GMFG	-0.0637	$3.80x10^{-10}$	0.26	0.37	NS	NS	NS	NS
cg02794990	15	59659880	MYO1E	-0.063	$5.09x10^{-4}$	NS	NS	-1.52	-0.59	NS	NS
cg10416593	22	50966123	TYMP	-0.063	$1.62x10^{-6}$	NS	NS	1.31	0.93	1.87	2.64
cg01059398	3	172235808	TNFSF10	-0.062	$2.71x10^{-45}$	NS	NS	0.97	1.96	NS	NS
cg21406967	7	100464553	TRIP6	-0.0616	$2.11x10^{-4}$	NS	NS	0.73	0.73	NS	NS
cg17744295	19	16178097	TPM4	-0.0613	$3.31x10^{-57}$	NS	NS	1.02	1.11	NS	NS

cg15667844	10	112256729	DUSP5	-0.0612	1.86×10^{-4}	NS	NS	NS	NS	2.18	2.39
cg04858586	1	174843971	RABGAP1L	-0.0609	6.77×10^{-4}	1.24	-0.46	NS	NS	NS	NS
cg16227684	x	153664862	GDI1	-0.0601	1.75×10^{-10}	-0.65	-0.42	NS	NS	NS	NS
cg01293485	3	42847044	HIGD1A	-0.0601	2.03×10^{-6}	0.57	0.48	NS	NS	NS	NS
cg10778971	14	94577101	IFI27	-0.0599	3.17×10^{-26}	6.82	5.71	5.83	5.69	6.09	7.99
cg17338424	11	46722416	ARHGAP1	-0.0598	3.36×10^{-5}	NS	NS	-0.37	-0.5	NS	NS
cg04835284	10	102279758	SEC31B	-0.0596	3.05×10^{-3}	NS	NS	-1.09	-0.4	NS	NS
cg13592947	5	1111049	SLC12A7	-0.0595	3.40×10^{-11}	-0.58	-0.58	NS	NS	NS	NS
cg26064470	5	32275566	MTMR12	-0.0591	3.33×10^{-10}	NS	NS	0.71	0.74	NS	NS
cg01305174	6	7313966	SSR1	-0.0585	4.92×10^{-4}	NS	NS	1.59	1.26	NS	NS
cg00356361	18	77286260	NFATC1	-0.0583	2.11×10^{-22}	NS	NS	-1.2	-0.48	NS	NS
cg15476723	19	48116808	GLTSCR1	-0.0582	5.01×10^{-4}	NS	NS	-0.42	-0.57	NS	NS
cg15659713	8	38586183	TACC1	-0.0573	7.01×10^{-5}	NS	NS	-0.35	-0.5	NS	NS
cg24419094	2	10266986	RRM2	-0.0565	2.42×10^{-10}	NS	NS	3.75	3.54	2.89	2.25
cg00431602	x	23799775	SAT1	-0.0565	4.19×10^{-4}	NS	NS	NS	NS	0.66	1.16
cg17886959	16	56642024	MT2A	-0.0564	9.14×10^{-12}	0.92	0.96	0.94	1.13	1.1	1.74
cg13127231	13	111806949	ARHGEF7	-0.0561	5.01×10^{-5}	-0.18	0.26	NS	NS	NS	NS
cg04103418	11	130076148	ST14	-0.0559	1.09×10^{-9}	NS	NS	-1.19	-1.5	NS	NS
cg14442492	5	95296150	ELL2	-0.0555	1.35×10^{-3}	NS	NS	3.26	1.3	NS	NS
cg01966878	4	90757139	SNCA	-0.0547	3.70×10^{-4}	NS	-1.13	NS	NS	NS	NS
cg14553895	3	178899170	PIK3CA	-0.0546	1.87×10^{-3}	1.14	1.34	NS	NS	NS	NS

Differential gene expression values calculated for SLE versus controls in CD14+ monocytes (GSE38351), CD19/20+ B cells (GSE10325, GSE4588), and CD4+ T cells (GSE10325, GSE51997). NS indicates not significant in differential expression experiment (FDR P-value > 0.2)

*CpGs with P<0.01 and $\Delta\beta < -0.055$.

†Positions are from Build 37.

‡Active disease is defined as >= 6 on the Systemic Lupus Erythematosus Disease Activity Index (SLEDAI).[35]

Table S4. Differentially methylated probes from three monozygotic twin pairs discordant for SLE.

CpG*	Chr	Pos (bp)†	Gene	$\Delta\beta$ mean	P-value	Interferon-Regulated‡	Relation to CpG††	iMETHYL Summary eQTM	iMETHYL Summary Cell: P-value
cg13304609	1	79085162	IFI44L	-0.29	$1.58x10^{-14}$	IRG		-	-
cg06872964	1	79085250	IFI44L	-0.24	$1.05x10^{-71}$	IRG		-	-
cg03607951	1	79085586	IFI44L	-0.26	$7.23x10^{-22}$	IRG		IFI44L	PBMC: P=$1.82x10^{-44}$
cg17515347	1	159047163	AIM2	-0.09	$3.01x10^{-12}$	IRG		TAGLN2<br>SLAMF8<br>DUSP23<br>PYHIN1<br>FCRL6	CD4+T: P=$3.09x10^{-5}$<br>CD4+T: P=$2.50x10^{-4}$<br>CD4+T: P=$1.90x10^{-5}$<br>CD4+T: P=$8.24x10^{-8}$<br>CD4+T: P=$3.62x10^{-6}$
cg08272268	1	200380059	ZNF281	-0.09	$4.33x10^{-15}$		S_Shore	-	-
cg01028142	2	7004578	CMPK2	-0.33	$7.98x10^{-8}$	IRG	N_Shore	-	-
cg10959651	2	7018020	RSAD2	-0.13	$3.14x10^{-14}$	IRG		-	-
cg10549986	2	7018153	RSAD2	-0.09	$1.95x10^{-91}$	IRG		-	-
cg14126601	2	37384708	EIF2AK2	-0.1	$5.55x10^{-16}$	IRG	S_Shore	EIF2AK2	PBMC: P=$5.66x10^{-11}$
cg26337070	2	85999873	ATOH8	-0.1	$7.55x10^{-9}$			-	-
cg04781494	2	202047246	CASP10	-0.09	$8.39x10^{-8}$	IRG		-	-
cg15768138	2	219030752	CXCR1	-0.11	$7.38x10^{-27}$			-	-
cg13411554	3	53700276	CACNA1D	-0.09	$8.66x10^{-8}$			-	-
cg22930808	3	122281881	PARP9-DTX3L	-0.37	$6.74x10^{-126}$	IRG	N_Shore	-	-
cg08122652	3	122281939	PARP9-DTX3L	-0.38	$1.11x10^{-9}$	IRG	N_Shore	-	-
cg00959259	3	122281975	PARP9-DTX3L	-0.34	$1.32x10^{-56}$	IRG	N_Shore	-	-
cg06981309	3	146260954	PLSCR1	-0.24	$6.41x10^{-31}$	IRG	N_Shore	-	-
cg02556393	3	168866705	MECOM	-0.09	$3.14x10^{-95}$		N_Shore	-	-
cg07809027	4	15007205	CPEB2	-0.1	$2.08x10^{-14}$		S_Shore	-	-
cg02215171	4	89379156	HERC5	-0.09	$4.48x10^{-18}$	IRG	S_Shore	HERC5	PBMC P=$5.18x10^{-8}$
cg17786255	4	108814389	SGMS2	-0.09	$2.01x10^{-16}$	IRG		-	-
cg21873524	4	190942744		-0.11	$1.03x10^{-55}$		Island	-	-
cg24740632	5	134486678		-0.12	$2.26x10^{-60}$			-	-
cg06012695	6	28770593		-0.11	$3.59x10^{-16}$			-	-
cg25138053	6	31368016		-0.09	$3.67x10^{-15}$		S_Shore	-	-
cg22708150	6	31649619	LY6G5C	-0.14	$1.05x10^{-19}$		N_Shore	-	-

cg07292773	6	156718177		0.1	$2.22x10^{-17}$		Island	-	-
cg12013713	7	139760671	PARP12	-0.12	$1.44x10^{-16}$	IRG	N_Shore	-	-
cg20190772	8	48572496	KIAA0146	-0.09	$1.40x10^{-8}$			-	-
cg14864167	8	66751182	PDE7A	-0.35	$1.21x10^{-9}$		N_Shelf	-	-
cg06102678	8	81491328		-0.09	$1.00x10^{-8}$		Island	-	-
cg12110437	8	144098888	LY6E	-0.2	$3.14x10^{-9}$	IRG	N_Shore	RP11-273G15.2	CD4T: $P=1.70x10^{-4}$
cg17555806	10	74448117		-0.09	$1.51x10^{-8}$		N_Shelf	-	-
cg02314339	10	91020653		-0.11	$1.72x10^{-08}$			LIPA	PBMC: $P=6.27x10^{-10}$
cg06188083	10	91093005	IFIT3	-0.25	$6.18x10^{-8}$	IRG		-	-
cg05552874	10	91153143	IFIT1	-0.26	$6.01x10^{-16}$	IRG		IFIT1	PBMC: $P=1.57x10^{-11}$
cg14910175	10	131840954		-0.09	$1.56x10^{-11}$		N_Shelf	-	-
cg10552523	11	313478	IFITM1	-0.13	$5.90x10^{-115}$	IRG	N_Shelf	RP11-326C3.12<br>RP11-326C3.14	CD4T: $P=2.03x10^{-4}$<br>PBMC: $P=9.74x10^{-3}$
cg20566897	11	313527	IFITM1	-0.1	$7.00x10^{-62}$	IRG	N_Shelf	ATHL1<br>CD151<br>ATHL1<br>RP11-326C3.7	PBMC: $P=4.29x10^{-3}$<br>CD4T: $P=3.14x10^{-4}$<br>PBMC: $P=8.67x10^{-4}$<br>PBMC: $P=2.79x10^{4}$
cg23570810	11	315102	IFITM1	-0.27	$1.43x10^{-18}$	IRG	N_Shore	IFITM1<br>IFITM3	PBMC: $P=3.07x10^{-9}$<br>PBMC: $P=9.83x10^{-3}$
cg03038262	11	315262	IFITM1	-0.25	$4.41x10^{-40}$	IRG	N_Shore	ATHL1<br>ATHL1	PBMC: $P=2.71x10^{-5}$<br>PBMC: $P=4.41x10^{-4}$
cg20045320	11	319555		-0.18	$4.85x10^{-17}$		S_Shore	-	-
cg17990365	11	319718	IFITM3	-0.16	$8.78x10^{-295}$	IRG	S_Shore	IFITM3<br>IFITM2<br>RP11-326C3.15<br>RP11-326C3.12	PBMC: $P=9.81x10^{-3}$<br>PBMC: $P=1.62x10^{-4}$<br>PBMC: $P=1.27x10^{-7}$<br>PBMC: $P=1.16x10^{-9}$
cg08926253	11	614761	IRF7	-0.17	$2.01x10^{-9}$	IRG	Island	-	-
cg12461141	11	5710654	TRIM22	-0.1	$6.35x10^{-25}$	IRG		-	-
cg23571857	17	6658898	XAF1	-0.1	$1.46x10^{-8}$	IRG		XAF1	PBMC: $P=2.05x10^{-5}$
cg04927537	17	76976091	LGALS3BP	-0.15	$2.77x10^{-10}$	IRG		LGALS3BP	CD4T: $P=2.16x10^{-3}$

cg25178683	17	76976267	LGALS3BP	-0.16	2.01×10^{-8}	IRG		LGALS3BP	Mono: $P=1.63\times10^{-6}$<br>Neu: $P=1.42\times10^{-5}$<br>PBMC: $P=8.74\times10^{-22}$<br>PBMC: $P=8.80\times10^{-20}$
cg16503797	18	19476805		-0.09	5.39×10^{-12}		N_Shore	-	-
cg15871086	18	56526595		-0.09	2.08×10^{-11}		N_Shelf	-	-
cg23352030	20	62198469	PRIC285	0.14	2.36×10^{-11}		Island	-	-
cg16785077	21	42791867	MX1	-0.11	8.45×10^{-27}	IRG	N_Shore	-	-
cg22862003	21	42797588	MX1	-0.31	1.62×10^{-25}	IRG	N_Shore	MX1	CD4T: $P=2.94\times10^{-6}$<br>Momo: $P=8.62\times10^{-5}$
cg26312951	21	42797847	MX1	-0.21	6.28×10^{-15}	IRG	N_Shore	AP001610.5<br>MX2<br>AP001610.5<br>MX2<br>MX1	PBMC: $P=1.49\times10^{-4}$<br>PBMC: $P=5.71\times10^{-3}$<br>PBMC: $P=2.84\times10^{-11}$<br>PBMC: $P=4.57\times10^{-5}$<br>PBMC: $P=2.91\times10^{-13}$
cg21549285	21	42799141	MX1	-0.47	6.59×10^{-13}	IRG	S_Shore	MX1	PBMC: $P=5.61\times10^{-37}$
cg05543864	22	24979755	GGT1	-0.09	1.44×10^{-45}			-	-
cg20098015	22	50971140	ODF3B	-0.21	9.88×10^{-83}	IRG	S_Shore	SCO2<br>CTA-384D8.36<br>TYMP<br>NCAPH2<br>ODF3B<br>KLHDC7B	PBMC: $P=1.69\times10^{-7}$<br>PBMC: $P=6.04\times10^{-8}$<br>PBMC: $P=2.42\times10^{-7}$<br>PBMC: $P=2.85\times10^{-3}$<br>PBMC: $P=2.68\times10^{-7}$<br>PBMC: $P=6.81\times10^{-4}$
cg05523603	22	50973101		-0.22	5.51×10^{-14}		S_Shelf	-	-
cg02247863	22	50983415		-0.09	2.51×10^{-13}		N_Shore	-	-

*CpGs meeting the $P_{FDR}<0.05$ threshold (equivalent to $P<1.06\times10^{-7}$) and having $|\Delta\beta| > 0.085$.

†Positions are from Build 37.

‡IRG as defined by Interferome[37]

http://imethyl.iwate-megabank.org/

Figure S1. Area under the receiver operating characteristic curve (AUC) calculated at regular intervals between 0 and -0.15 in four cell types.

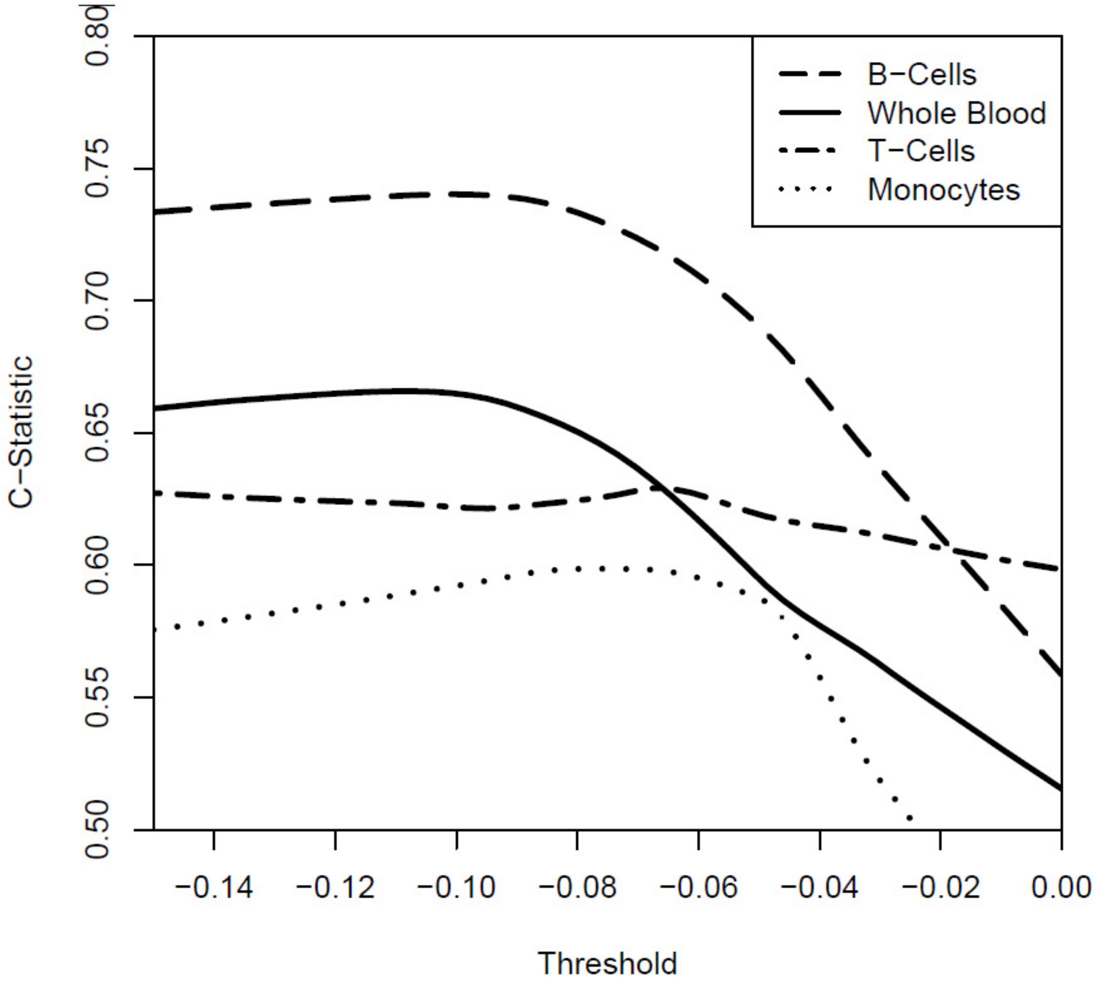

Figure S2. Proportions of significantly associated CpGs (as defined in Table 1) located in islands, shores, shelves, and other content categories.

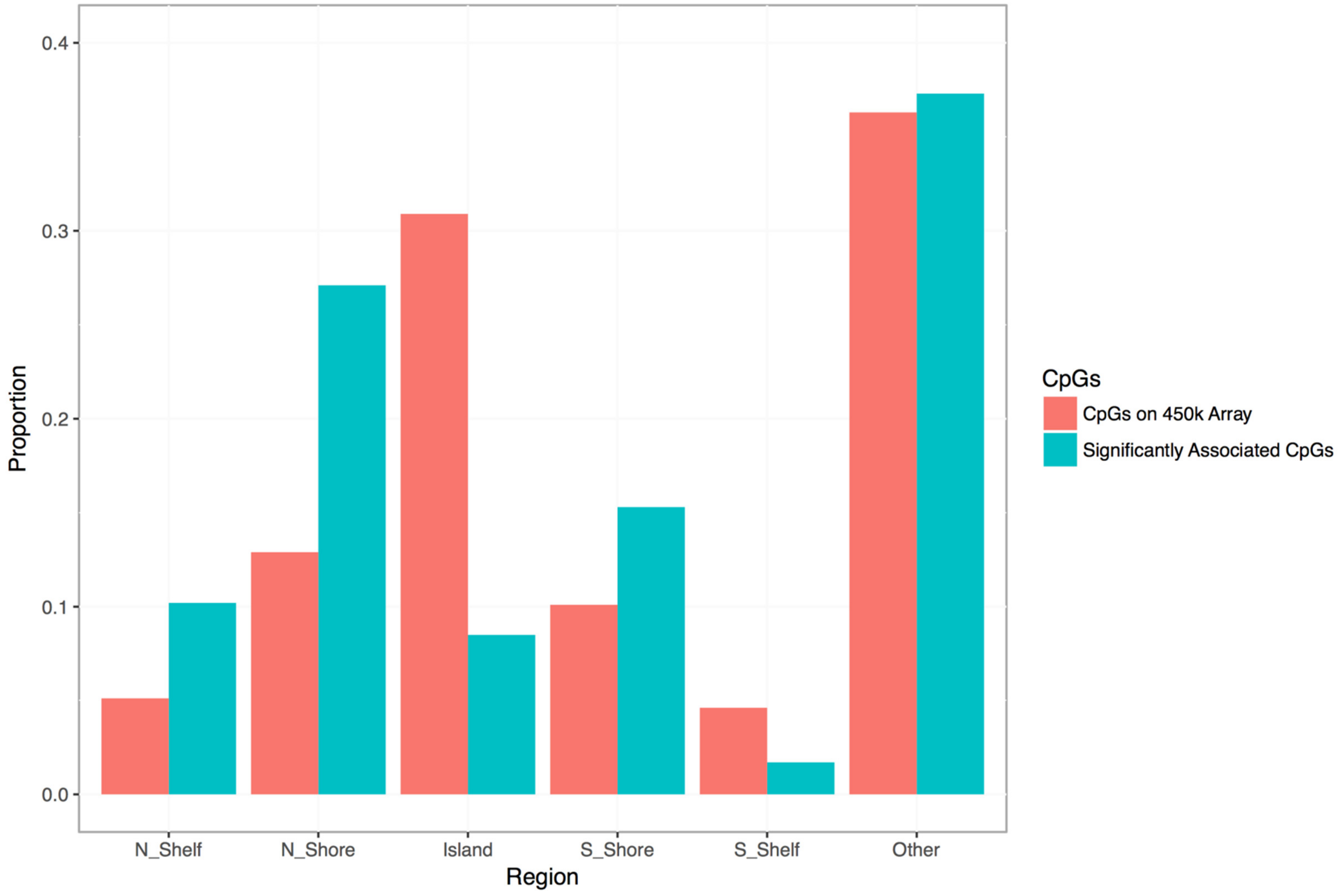

Supplement: Supplementary file 1 [file genes-12-01898-s001.zip › genes-1463577-Figure S1-2 and Table S1-4.pdf]
